# Molecular Evolution of the Primate α-/θ-Defensin Multigene Family

**DOI:** 10.1371/journal.pone.0097425

**Published:** 2014-05-12

**Authors:** Dong-Qiang Cheng, Ying Li, Jing-Fei Huang

**Affiliations:** 1 State Key Laboratory of Genetic Resources and Evolution, Kunming Institute of Zoology, Chinese Academy of Sciences, Kunming, China; 2 Kunming College of Life Science, University of Chinese Academy of Sciences, Beijing, China; 3 Institute of Animal Genetics and Breeding, College of Animal Science and Technology, Sichuan Agricultural University, Yaan, China; 4 Kunming Institute of Zoology-Chinese University of Hongkong Joint Research Center for Bio-resources and Human Disease Mechanisms, Kunming, China; University of Central Florida College of Medicine, United States of America

## Abstract

The primate α-/θ-defensin multigene family encodes versatile endogenous cationic and amphipathic peptides that have broad-spectrum antibacterial, antifungal and antiviral activity. Although previous studies have reported that α-/θ-defensin (*DEFA*/*DEFT*) genes are under birth-and-death evolution with frequent duplication and rapid evolution, the phylogenetic relationships of the primate *DEFA*/*DEFT* genes; the genetic bases for the existence of similar antimicrobial spectra among closely related species; and the evolutionary processes involved in the emergence of cyclic θ-defensins in Old World monkeys and their subsequent loss of function in humans, chimpanzees and gorillas require further investigation. In this study, the *DEFA/DEFT* gene repertoires from primate and treeshrew were collected, followed by detailed phylogenetic, sequence and structure, selection pressure and comparative genomics analyses. All treeshrew, prosimian and simian *DEFA*/*DEFT* genes are grouped into two major clades, which are tissue-specific for enteric and myeloid defensins in simians. The simian enteric and myeloid α-defensins are classified into six functional gene clusters with diverged sequences, variable structures, altered functional constraints and different selection pressures, which likely reflect the antimicrobial spectra among closely related species. Species-specific duplication or pseudogenization within each simian cluster implies that the antimicrobial spectrum is ever-shifting, most likely challenged by the ever-changing pathogen environment. The *DEFT* evolved from the myeloid *DEFA8*. The prosegment of θ-defensin is detected with adaptive changes coevolving with the new protein fold of mature peptide, coincident with the importance of the prosegment for the correct folding of the mature peptide. Lastly, a less-is-hitchhiking hypothesis was proposed as a possible explanation for the expansion of pseudogene *DEFTP* and the loss of functional *DEFT*, where the gain or loss of the hitchhiker is determined by its adjacent driver gene during the birth-and-death evolutionary process.

## Introduction

Host immune systems evolve various defensive weapons against pathogens during the pathogen-host arms race. Defensins are small antimicrobial peptides that act as both effectors and mediators of host immunity. These endogenous cationic and amphipathic peptides have broad-spectrum antibacterial, antifungal and antiviral activity [1]–[6]. They also modulate the innate and adaptive immune systems by promoting or suppressing the proinflammatory responses during microbial infection [7]–[9]. In mammals, there are three structural subfamilies of defensins—α-, β- and θ-defensins—that differ by their tridisulfide motifs. The β-defensins are the oldest members and are found in most classes of vertebrates [10]–[13]. The α-defensins, which originate from the β-defensins, are younger members. The α-defensins are found in marsupials and most mammals but were lost in some Laurasiatheria species, such as cattle and dogs [14]–[17]. The θ-defensins are the only mammalian cyclic peptides currently known and originate from α-defensins through a nonsense mutation in the mature peptide. The θ-defensins are found in Old World monkeys and certain apes but underwent a loss of function in humans, chimpanzees and gorillas due to a nonsense mutation in the signal peptide [18], [19].

Primate α-/θ-defensin (*DEFA/DEFT*) genes are located as multigene clusters on chromosome regions that are homologous with human chromosome 8p23 [16], [20]. Like other multigene families, *DEFA/DEFT* genes can be constitutively expressed at high levels to produce variant functional proteins. In humans, there are six functional and tissue-specific α-defensin peptides. Human HNP1–HNP4, encoded by the *DEFA1*, *DEFA3* and *DEFA4* genes, are primarily expressed in neutrophils [1]. The *DEFA1* and *DEFA3* genes are genetic variants that encode proteins with a single amino acid difference and are also referred to as *DEFA1A3* genes. Unlike the single-copy *DEFA4*, the *DEFA1A3* genes have copy number polymorphisms [21]–[23]. In contrast, human HD5 and HD6, encoded by *DEFA5* and *DEFA6*, respectively, are expressed primarily in Paneth cells of the small intestine and play important roles in intestinal host defense and homeostasis [24]–[26]. Apart from the functional genes, there are many defensin pseudogenes—*DEFAP* and *DEFTP*—in humans [19], [27]. In macaques, the new member θ-defensin encoded by *DEFT* is primarily expressed in the bone marrow and leukocytes [28].

Defensins are synthesized as pre-pro-defensins containing a signal peptide, a prosegment and a mature peptide. The prosegment, which serves as an intramolecular chaperone, assists in the correct disulfide pairing and proper folding of the mature peptide [29] and keeps the mature peptide inactive until it is cleaved by various proteolytic enzymes [30]–[32]. The mature peptides are cationic and amphipathic, which are important properties for inducing the depolarization and permeabilization of the microbial membrane [2], [33]. The α-defensin monomer has a three-stranded antiparallel β-sheet structure with three intramolecular disulfide pairs linked as Cys1-Cys6, Cys2-Cys4 and Cys3-Cys5. Two monomers form an amphipathic dimer, which is stabilized by hydrophobic interactions and intermolecular hydrogen bonds between residues 18 and 20 (HNP4 numbering) in the second β-sheet [33], [34]. The dimerization of α-defensins, in addition to their cationic and amphipathic character, is also important for their antimicrobial ability [35]–[37]. In contrast to the structure of α-defensins, the θ-defensins form a cyclic octadecapeptide through the posttranslational head-to-tail ligation of two nonapeptides and harbor three intermolecular disulfide pairs [18]. Recently, synthetic defensins have been studied and are being developed as potential antimicrobial peptide drugs [38]–[41].

Because of the frequent duplication and rapid evolution of primate α-/θ-defensins, the nomenclature and phylogenetic relationships among this multigene family are still ambiguous. Moreover, there is no clear phylogenetic classification related to the expression pattern or the confounding antimicrobial function of these α-defensins, although many functional studies indicate that α-defensins are effective microbicidal peptides against a wide variety of microorganisms. Previous studies have demonstrated that the α-/θ-defensin multigene family, like many other multigene families, is subject to birth-and-death evolutionary process with frequent gene duplication, pseudogenization and significant positive selection [42]–[44]. However, the molecular evolution of the undocumented antimicrobial spectra that are composed of functionally divergent α-/θ-defensins in humans and closely related primates should be further explored. In this study, the phylogenetic classification, sequence divergence and structural diversification of the primate α-/θ-defensins were investigated using molecular evolution and molecular dynamics analyses. Furthermore, the evolutionary processes involved in the emergence of cyclic θ-defensins and their subsequent loss of function in humans, chimpanzees and gorillas require investigation. Loss of function is a major driving force for phenotypic change and can be advantageous, deleterious or tolerated, as explained by the hypotheses of less-is-more, less-is-less and less-is-nothing, respectively [45]. Because of the frequent gene duplication and functional redundancy within multigene families, losing certain members is usually “tolerable”. However, the θ-defensins have significant antiviral activity that is higher than that of α-defensins and β-defensins, including anti-HIV-1 activity [39], [46]. The loss of functional θ-defensin seems to be “deleterious”. Therefore, the expansion of pseudogene *DEFTP* and the loss of functional *DEFT* in humans, chimpanzees and gorillas cannot be well explained by the three hypotheses noted above. The evolutionary fate of θ-defensin genes may be influenced by other factors, which should be further investigated. In this study, a less-is-hitchhiking hypothesis was proposed to explain this process, using comparative genomics analyses.

## Materials and Methods

### Sequence identification

To identify sequences for the primate α-/θ-defensin gene family, a literature search and exhaustive genome blast searches were carried out. First, primate *DEFA*/*DEFT* sequences were collected from the literature and defined as queries. Second, sequence-based searches of both BlastN and BLAT were performed using these queries, and these BlastN and BLAT searches were repeated using the newly identified genes as queries until no additional genes could be identified. Last, all identified genes were manually checked for functional genes or pseudogenes. Primate *DEFA/DEFT* DNA sequences or completely assembled genomes from the NCBI database or the UCSC Genome Browser were used, including human (*Homo sapiens*; assembly: GRCh37.p5; March 2011), chimpanzee (*Pan troglodytes*; assembly: Pan_troglodytes-2.1.4; March 2011), gorilla (*Gorilla gorilla*; assembly: gorGOR3.1; October 2011), Sumatran orangutan (*Pongo abelii*; assembly: AC206038.3; August 2008), northern white-cheeked gibbon (*Nomascus leucogenys*; assembly: Nleu1.0; October 2011), siamang (*Symphalangus syndactylus*; sequences: AY128121.1 and AY128122.1), macaque (*Macaca mulatta*; assembly: rheMac2; January 2006), crab-eating macaque (*Macaca fascicularis*; assemblies: AEHL01390702.1 and CAEC01613298.1; October 2011), olive baboon (*Papio anubis*; assembly: AC116559.30; September 2010), pig-tailed macaque (*Macaca nemestrina*; AY128123.1), eastern black-and-white colobus (*Colobus guereza kikuyuensis*; AY128124), marmoset (*Callithrix jacchus*; assembly: calJac3; June 2007), Bolivian squirrel monkey (*Saimiri boliviensis boliviensis*; assembly: saiBol1; November 2011), gray mouse lemur (*Microcebus murinus*; assembly: ABDC01305195.1; July 2007), bush baby (*Otolemur garnettii*; assembly: AAQR03188273.1; March 2011), and Philippine tarsier (*Tarsius syrichta*; assembly: ABRT010372935.1; September 2008). The treeshrews (northern treeshrew *Tupaia belangeri*; assembly: AAPY01804345.1; June 2006; Chinese treeshrew *Tupaia belangeri chinensis*; assembly: ALAR1000000; February 2013), which are close relatives of primates, were also used. The exon-intron structures were determined by GeneWise. Sequences that encoded α-/θ-defensins containing the six conserved cysteines (for *DEFA*) and the three conserved cysteines (for *DEFT*) were identified as functional genes. Sequences with frame-shift (insertion or deletion) mutations or premature stop codons were considered pseudogenes. A list of identified sequences is detailed in Table S1.

### Molecular evolutionary analyses

To understand the evolutionary processes of the primate α-/θ-defensin gene family, molecular evolutionary analyses and comparative genomics analyses were performed. Coding regions of the *DEFA*/*DEFT* functional genes were aligned codon-to-codon using the MUSCLE program as implemented in the MEGA6 software [47], and then corresponding regions of the pseudogenes were manually matched to the multiple sequence alignment of the functional genes.

Phylogenetic trees were built using either the more conserved signal-prosegment region or the entire coding region. To obtain reliable phylogenetic relationships, three different tree-building methods were combined. Bayesian inference (BI) phylogenetic trees were constructed using the BEAST software [48]. Neighbor-joining (NJ) and maximum likelihood (ML) phylogenetic trees were constructed using the MEGA6 software [47]. The BI trees were computed using the general time-reversible (GTR) nucleotide substitution model, and the site heterogeneity model was assumed with a discrete gamma distribution for among-site rate variation and a proportion of invariant sites (G + I (4 categories)). Two independent Markov chain Monte Carlo (MCMC) runs were conducted for 10,000,000 steps, with a sampling frequency of every 1000 steps, a UPGMA starting tree and other default parameters. When the effective sample sizes (ESSs) of all quantities were larger than 200, the MCMC was considered converged, and the results were analyzed with 20% burn-in. The best nucleotide substitution patterns for ML trees were selected based on the analyses of best-fit models in MEGA6. The ML trees were computed using the Kimura 2-parameter substitution model (K2) with a bootstrap test (1000 replicates), and the rate variation model was allowed with a discrete gamma distribution (4 categories). The evolutionary distances for NJ trees were computed using the Kimura 2-parameter method with a bootstrap test (1000 replicates), and the rate variation among sites was modeled with a gamma distribution (gamma parameter  = 2.6 for the signal-prosegment region and 1.4 for the entire coding region).

The phylogenetic trees of BI, NJ and ML were combined using the TreeGraph2 software [49]. The BI trees were selected as background trees, and the clades or clusters of similar topologies in the BI, NJ and ML trees were labeled with posterior probabilities (BI) and bootstrap support values (NJ and ML).

The *DEFA/DEFT* genes from the human, chimpanzee, orangutan, macaque and marmoset genomes were used for synteny analysis by mapping their loci onto chromosomes in scale.

Functional divergences (type I and type II) between clusters were tested using the Gu99 and Gu2013 probabilistic models implemented in the DIVERGE 2.0 software [50], [51]. Pairwise coefficients (θ*_ij_*±SE) and likelihood ratio statistics (LRT) for simian enteric or myeloid defensin clusters were analyzed using the bootstrapped Gu99 probabilistic method. Functional distance analysis was performed based on the Wang and Gu 2001 method [52]. The type I functional distance (*d*_F_) between clusters was defined as *d*_F_ = −ln(1−θ*_ij_*). For two clusters A and B, *d*_F_(A,B) = *b*_F_(A)+*b*_F_(B); thus, the functional branch length, *b*_F_, for each cluster can be calculated using a standard least-squares method. A large *b*_F_ value for a gene cluster indicates that the evolutionary conservation may be shifted at many sites. The effective number of sites (n_e_) related to functional divergences was estimated following the Gu2013 protocol. Type I sites, typically generated by type I functional divergence, are conserved in one gene cluster but highly variable in the other following gene duplication, whereas type II sites, which are primarily caused by type II functional divergence, refer to radical changes of conserved amino acids at the same site in both gene clusters.

To investigate the selection pressure, maximum likelihood estimations of positive selection were performed using the site-specific models from the CODEML program implemented in the PAML 4.5 package [53]. Parameters for the models M0 (one ratio), M1a (neutral), M2a (selection), M7 (beta), M8 (beta and ω (equivalent to Ka/Ks)) and M8a (beta and ω = 1) were calculated. The M0 model assumes a uniform selective pressure among sites. The M1a model assumes a variable selective pressure but no positive selection. The M2a model assumes a variable selective pressure with positive selection. The M7 model assumes a beta-distributed variable selective pressure. The M8 model assumes a beta-distributed variable selective pressure plus positive selection. The M8a assumes a beta distributed variable selective pressure without positive selection. Three likelihood ratio tests (LRTs) were compared using the following paired models: M1a-M2a, M7-M8 and M8a-M8.

To investigate changes in the selection pressure along the *DEFA* and *DEFT* sequences, amino acid identities and average pairwise Ka/Ks ratios were computed using a sliding window method with a window size of 10 residues and a step size of 5 residues. Within each window, the numbers of nonsynonymous and synonymous substitutions per site (denoted as Ka and Ks, respectively) were calculated using the Nei and Gojobori (1986) method incorporated into the KaKs_Calculator software [54]. The values of Ka/Ks were reanalyzed using a bootstrap test of 1000 replicates to assess the effect of potential biases from a few genes in the gene set and to test the statistical significance of the difference between the average Ka/Ks ratio and 1. Suppose there were N original sequences, the average Ka/Ks from a random resampling of N sequences was calculated, and the resampling process was repeated 1000 times. The histogram for the bootstrap estimates of average Ka/Ks showed a normal distribution, and the parameters of the normal distribution were calculated. The null hypothesis (H0) was that there was no difference between Ka/Ks and 1, and the alternative hypothesis (H1) was that there was a difference between Ka/Ks and 1. If the average Ka/Ks for the original sequences was larger than 1, the H0 was defined as “Ka/Ks≤1” and the H1 was defined as “Ka/Ks>1.” If the average Ka/Ks for the original sequences was smaller than 1, the H0 was defined as “Ka/Ks≥1” and the H1 was defined as “Ka/Ks<1.” The p-value was calculated based on the parameters of the normal distribution.

Sequence logos were generated from the web server WebLogo [55].

### Molecular dynamics simulations

Structures of the α- and θ-defensins were searched using BlastP against the Protein Data Bank (PDB) database. One representative structure for each simian α-defensin cluster was analyzed, including human HNP1 (PDB code: 3GNY) [56] representing the DEFA1 cluster, human HNP4 (PDB code: 1ZMM) for the DEFA4 cluster, human HD5 (PDB code: 1ZMP) for the DEFA5 cluster and human HD6 (PDB code: 1ZMQ) for the DEFA6 cluster [34]. No native structures were solved for the DEFA8 and DEFA9 clusters; thus, homology models of the rhesus macaque DEFA8 and marmoset DEFA9a were built using the MODELER automodel method [57] implemented in the Discovery Studio 3.1 software. The corresponding template structures selected for this modeling were HNP1 (41.9% sequence identity with DEFA8) and HD5 (39.4% sequence identity with DEFA9). Monomers were aligned into dimers by superimposing four intermolecular hydrogen bonds between the backbone residues 18 and 20 (HNP4 numbering) through a customized TCL script running in the VMD 1.9 software [58].

Molecular dynamics (MD) simulations were performed following the method described below. The simulation system was parameterized by applying the standard AMBER force field ff99SB for bio-organic systems using the leap module implemented in the AmberTools 12 package [59]. The preparative processes included adding hydrogen atoms and disulfide bonds, neutralizing with counter ions (Cl-) and solvating in a periodic box with TIP3P water to at least a 10-Å distance around the protein. Molecular dynamics simulations were then performed using the NAMD 2.7 software [60]. The entire simulation process was composed of the following steps: minimization, heating, equilibration and production. Three rounds of energy minimization were performed by releasing the restraints in a stepwise fashion. In the first round, all protein atoms were constrained for 50,000 steps with 2.0 kcal/(mol•Å^2^) restraints. In the second round, only atoms of the backbone were restrained for 50,000 steps with 2.0 kcal/(mol•Å^2^) restraints. In the third round, all atoms were relaxed for 100,000 steps. Short-range electrostatic and Van der Waals interactions were truncated at 9 Å, whereas long-range electrostatic forces were computed using the particle-mesh Ewald (PME) summation method. Next, the system was gently annealed from 0 to 310 K using a Langevin thermostat with a coupling coefficient of 5.0 ps^−1^ and employing a force constant with 2.0 kcal/(mol•Å^2^) restraints. All subsequent steps were carried out in the isobaric-isothermal NPT ensemble with a target pressure of 1 atm. Five rounds of equilibration (100 ps, each at 310 K) were performed with decreasing restraint weights from 2.0, 1.5, 1.0, 0.5 to 0.0 kcal/(mol•Å^2^). By releasing all the restraints, the system was again equilibrated for 500 ps. After the final equilibration step, the production phase of MD was run without any restraints for a total of 16 ns.

Tertiary structures and electronic properties were analyzed after performing MD simulations. The flexibility of the dimer was evaluated using two distances and a dihedral angle involving four highly flexible residues. The distance between the C^α^ atoms of residue 22 in each dimer (HNP4 numbering) was defined as A22C^α^-B22C^α^. The distance between the C^α^ atoms of residue 11 in each dimer was defined as A11C^α^-B11C^α^. The dihedral angle was defined as A11C^α^-A22C^α^-B22C^α^-B11C^α^. Distances and dihedral angles were extracted using a customized TCL script program.

The electrostatic potential was mapped by averaging the last five frames of each MD trajectory using the APBS plugin [61] implemented in the VMD software.

## Results

### Identification, nomenclature and phylogeny of the primate α-/θ-defensin multigene family

The primate α-/θ-defensin gene repertoires, which were identified from the published literature [19], [42], [62] and exhaustive genomic BlastN and BLAT searches, were manually checked. A total of 144 *DEFA/DEFT* sequences from primates were identified, including 105 functional genes and 39 pseudogenes. Moreover, 10 *DEFA* sequences from two species of treeshrews were collected and served as closely related outgroups for the primate sequences (Table S1). Almost one-third of the primate α-/θ-defensin genes have lost their functional protein-coding ability because of nonsense or frame-shift mutations that have occurred during the process of rapid birth-and-death evolution. The translated amino acid sequences of functional genes were aligned with MUSCLE (Figure S1). Most of the protein sequences are computationally translated from genome sequences rather than being demonstrated to be produced in vivo. The multiple sequence alignment shows that the mature peptide of α-defensins has a motif with six conserved cysteine residues, represented as C-x-C-x(3,4)-C-x(9)-C-x(6,9)-C, whereas the mature peptide of θ-defensins has a nonapeptide motif with three conserved cysteine residues, represented as x-C-x-C-x(4)-C, with the exception of the Eastern black-and-white colobus θ-defensin (*cgue_DEFT*), which has the motif x-C-x-C-x(8)-C.

**Figure 1:**
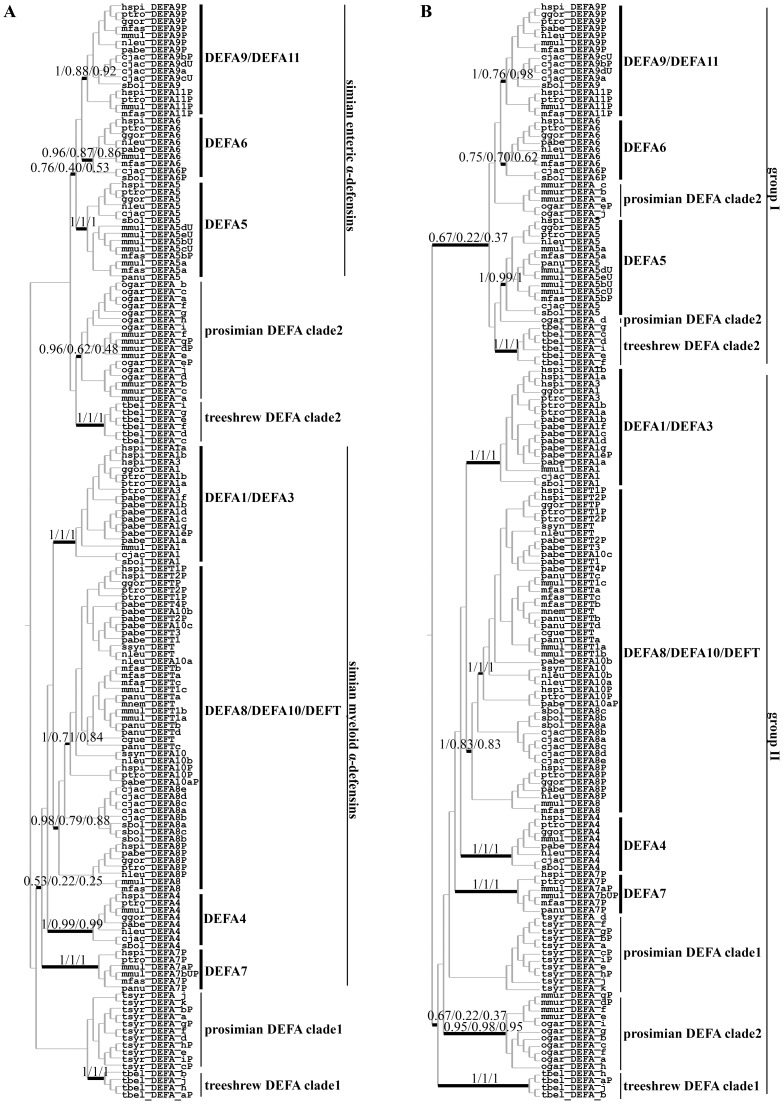
Phylogenetic trees of the α-/θ-defensin (*DEFA*/*DEFT*) genes in primates and treeshrews. A: Phylogenetic tree of primate and treeshrew *DEFA/DEFT* genes based only on the signal-prosegment region. The BI tree is selected as the background tree. The major clades or clusters having similar topologies from all three tree-building methods (BI, NJ and ML) are combined and labeled with the BI posterior probabilities and the bootstrap support values from the NJ and ML analyses. Primate *DEFA/DEFT* genes are clustered into prosimian and simian clades. The simian clades are classified and named following the nomenclature used for the human *DEFA/DEFT* genes. The “P” in node labels denotes a pseudogene, and the “U” indicates a gene with an ambiguous locus from the species in the synteny map in Figure 7. B: Phylogenetic tree of primate and treeshrew *DEFA/DEFT* genes based the entire coding region. The BI tree is selected as the background tree. The treeshrew *DEFA* genes are the outgroups of the two separate clades.

To explore the evolutionary relationships among these defensin genes, phylogenetic trees were constructed based on nucleotide sequences from either the more conserved signal-prosegment region or the entire coding region, and the phylogenetic trees inferred by the BI, NJ and ML methods were combined (Figure 1). All sequences are classified into major clades, which are referred to as the treeshrew, prosimian and simian clades based on the species included. In all the trees from the signal-prosegment region (Figures 1A, S2 and S3), the simian defensins are classified into two major clades of tissue-specific expression profiles and are thus named simian myeloid α-defensins and simian enteric α-defensins. In all the trees from the entire coding region (Figures 1B, S4 and S5), all sequences are separated into two groups, with group I containing the simian enteric α-defensins and the treeshrew clade 2 and group II containing the simian myeloid α-defensins and the treeshrew clade 1, which indicates a possible early duplication and divergence before the split of treeshrews and primates. However, for the prosimian clades, phylogenetic incongruence was observed. The tree inferred from the signal-prosegment region has two major prosimian clades (Figure 1A), whereas the tree inferred from the entire coding region divides the prosimian clade 2 into four groups (Figure 1B). Because incongruence between phylogenetic trees may result from the independence of evolutionary changes at different sites and the homogeneity of the substitution process [63], the phylogenetic incongruence was further assessed by reconstructing the phylogenetic tree after removing the homogeneity sites. Some amino acid sites in the mature peptide were identified as homogeneity sites of convergent or parallel evolution (Figure S6). When those sites were removed, the prosimian α-defensins grouped together, suggesting that these convergent or parallel evolution sites have resulted in long-branch attraction such that distant clades grouped together.

The simian myeloid and enteric α-defensins are classified into seven clusters, which are named following the nomenclature used for human *DEFA/DEFT* genes. The simian myeloid α-defensins contain the *DEFA1/DEFA3*, *DEFA8/DEFA10/DEFT*, *DEFA4* and *DEFA7* clusters, and the simian enteric α-defensins contain the *DEFA9/DEFA11*, *DEFA6* and *DEFA5* clusters. The *DEFA7* cluster contains only pseudogenes and is found only in Old World monkeys and hominoids. The other six gene clusters, each containing at least one functional gene, are functional gene clusters. All six functional gene clusters include species from New World monkeys, Old World monkeys and hominoids, suggesting that the duplication and diversification of these clusters occurred before the species differentiation of simians. The six functional gene clusters, which diverged prior to the simian differentiation, provide the genetic bases for the similar antimicrobial spectra in closely related simian species.

The *DEFA9/DEFA11* cluster shows different topologies among the phylogenetic trees (Figures S2 and S4). In the NJ and ML trees from the signal-prosegment region, the pseudogene *DEFA11P* and the *DEFA9* from Old World monkeys and hominoids group into one cluster, and the *DEFA9* from New Old monkeys is the outgroup. However, in the BI tree from signal-prosegment region and the BI/NJ/ML trees from the entire coding region, *DEFA11P* and *DEFA9* form separate clusters. To further clarify the relationship between *DEFA9* and *DEFA11P*, the sequence alignment from the complete gene including the intron was examined, and different patterns of sequence similarity were observed before and after a clear boundary in the genes. Therefore, phylogenetic trees were inferred based on either the sequences before the boundary (part I) or the sequences after the boundary (part II), using *DEFA6* as the outgroup (Figure S7). The trees based on part I show that *DEFA11P* is duplicated from *DEFA9* after the split of New World and Old World monkeys, whereas the trees based on part II show that *DEFA11P* is the outgroup of all *DEFA9*. The different topologies indicate that the two parts of *DEFA11P* might be derived from different ancestors. The *DEFA8* from Old World monkeys and hominoids also shows different phylogenetic positions among the phylogenetic trees (Figures S2 and S4). In the NJ tree from the signal-prosegment region, the *DEFA8* from Old World monkeys and hominoids clusters together with *DEFA10/DEFT*. However, in all the other trees, the *DEFA8* from Old World monkeys and hominoids is the outgroup in the *DEFA8/DEFA10/DEFT* cluster. Similarly, different patterns of sequence similarity were observed before and after a clear boundary in the genes of this cluster. Phylogenetic trees suggest that part I and part II of the *DEFA8* from Old World monkeys and hominoids might be derived from different ancestors (Figure S8). The complicated phylogenetic relationships within both the *DEFA9/DEFA11* cluster and the *DEFA8/DEFA10/DEFT* cluster imply that gene conversion or interlocus recombination might have occurred during the evolutionary process.

Unlike the *DEFA* genes, which are present in all primates, *DEFT* genes are present in only Old World monkeys and certain hominoids. Both the tree inferred from the entire coding region and the tree inferred from the signal-prosegment region show that *DEFT* groups together with *DEFA10*. *DEFA10*, which is present only in hominoids, is the basal clade in the *DEFA10/DEFT* cluster (Figure 1). The relationship between *DEFA10* and *DEFT* and the origin of *DEFT* were further analyzed using their introns. The BI/NJ/ML trees from the introns support that *DEFT* and *DEFA10* are grouped into separate clusters, and both clusters are derived from the duplication of *DEFA8* (Figure S9).

### Sequence divergence and structural variation of simian α-defensins

After identifying different clusters of primate α-/θ-defensins, the pattern of natural selection operating on each cluster was examined using the site-specific models of heterogeneous selection pressure among sites. Likelihood ratio tests (LRTs) were carried out between models M1a (neutral) and M2a (selection), M7 (beta) and M8 (beta and ω (equivalent to Ka/Ks)), and M8a (beta and ω = 1) and M8 [53]. The models M2a and M8, which are significantly favored over the other models, indicate positive selection, and the positively selected sites with posterior probabilities greater than 0.95 are listed (Table 1 and S2). Positive selection is not significantly detected for every cluster. For the two prosimian clades and the simian *DEFT*, *DEFA1* (including *DEFA3*), *DEFA5* and *DEFA8* (including *DEFA10*) clusters, positively selected sites are detected and are located primarily in the mature peptide. However, the simian *DEFA4*, *DEFA6* and *DEFA9* clusters are under purifying selection, and no positively selected site is detected. The different selection patterns among these clusters reflect their variant functional constraints. Therefore, the sequence divergence and structural variations among the six clusters (excluding the *DEFA7* pseudogene cluster) were further investigated to understand their functional diversity, which most likely constitutes their antimicrobial spectra.

**Table 1:** Log-likelihood values and positively selected sites for primate *DEFA/DEFT* genes under site models.

Cluster	Models (2ΔlnL)	Positively Selected Sites	P value
DEFT (n = 16)	M1a versus M2a (12.57)	67I 69R	<0.001
	M7 versus M8 (12.60)	17Q 50D 67I **69R**	0.002
	M8a versus M8 (12.58)		<0.001
DEFA1 (n = 16)	M1a versus M2a (22.42)	29V **67Y** **74I**	<0.001
	M7 versus M8 (23.33)	29V **67Y** **74I** 86Q	<0.001
	M8a versus M8 (22.40)		<0.001
DEFA4 (n = 8)	M1a versus M2a (3.02)		0.082
	M7 versus M8 (3.32)		0.190
	M8a versus M8 (3.02)		0.082
DEFA8 (n = 15)	M1a versus M2a (119.67)	**65R 67I 70R** 71G **72I 75L 76L 79R 80Y 82S 84A 85F 87G 92I**	<0.001
	M7 versus M8 (121.57)	**65R 67I 70R** 71G **72I 75L 76L 79R 80Y 82S 84A 85F 87G 92I**	<0.001
	M8a versus M8 (119.66)		<0.001
DEFA5 (n = 13)	M1a versus M2a (59.78)	**68T 70R** 72A **73T 74R** 77L **80V 82E 83I 84S**	<0.001
	M7 versus M8 (63.85)	25R 62A **68T 70R** 72A **73T 74R** 77L **80V 82E 83I 84S**	<0.001
	M8a versus M8 (59.72)		<0.001
DEFA6 (n = 7)	M1a versus M2a (3.33)		0.068
	M7 versus M8 (3.35)		0.187
	M8a versus M8 (3.32)		0.068
DEFA9 (n = 4)	M1a versus M2a (2.51)		0.113
	M7 versus M8 (2.52)		0.284
	M8a versus M8 (2.50)		0.114
prosimian DEFA clade1 (n = 6)	M1a versus M2a (16.11)	69I 79R **83V** 87R	<0.001
	M7 versus M8 (16.84)	66H 69I 79R **83V** 87R	<0.001
	M8a versus M8 (16.32)		<0.001
prosimian DEFA clade2 (n = 14)	M1a versus M2a (76.36)	**66R 73G** 79Y **87F** **90L**	<0.001
	M7 versus M8 (79.40)	37T **66R** 71R **73G** 74F 78T **79Y 87F 90L**	<0.001
	M8a versus M8 (72.84)		<0.001

Notes: positively selected sites with significance at the 99% level are bold, whereas the remaining sites have significance at the 95% level. The likelihood ratio tests are analyzed by comparing the following pairs of models: M1a-M2a, M7-M8 and M8a-M8.

**Figure 2:**
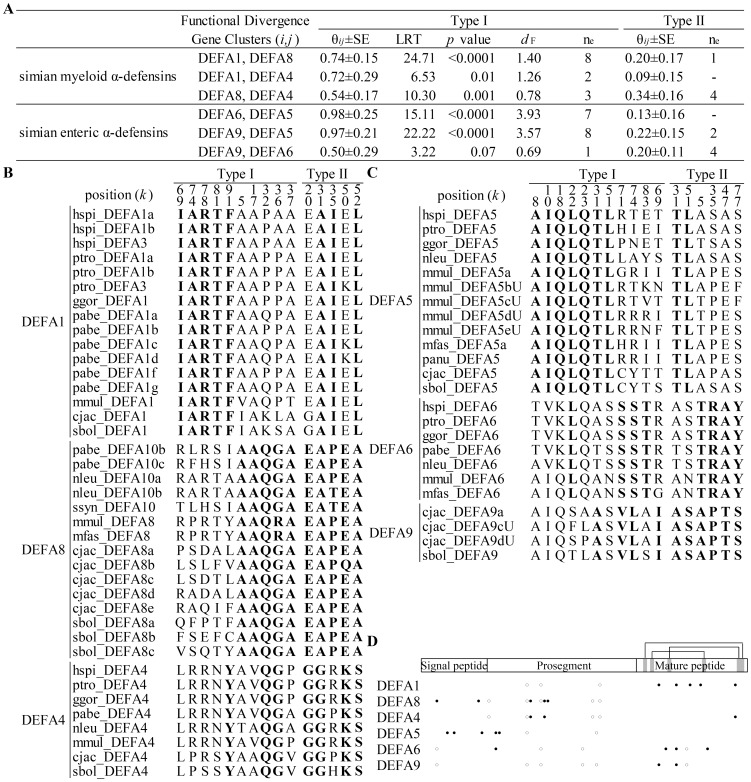
Type I and type II functional divergences among simian enteric or myeloid α-defensin clusters. A: Statistical analyses of the type I and type II functional divergences. Clusters within both simian enteric α-defensins and simian myeloid α-defensins have diverged. Pairwise coefficients (θ*_ij_*±SE) and likelihood ratio statistics (LRT) are analyzed using the bootstrapped Gu99 method. Type I functional distance (*d*_F_) between clusters is calculated as *d*_F_ = −ln(1−θ*_ij_*). The effective number of functional divergence related sites (n_e_) is estimated following the Gu2013 method. B: Critical amino acid sites responsible for type I and type II functional divergences from simian myeloid α-defensin clusters. These sites are identified based on the n_e_ cutoff after ranking posterior probabilities. C: Critical amino acid sites responsible for type I and type II functional divergences from simian enteric α-defensin clusters. D: The diagram of the sites responsible for type I and type II functional divergences in different simian clusters. Closed circles represent sites responsible for type I functional divergence, and opened circles denote sites responsible for type II functional divergence.

During the evolutionary process following gene duplication, due to functional divergence, the evolutionary rate of duplicated genes will initially increase and may subsequently shift with altered functional constraints (type I functional divergence) or remain at the original rate with no altered functional constraints (type II functional divergence) [50], [51], [64]. Type I and type II functional divergence patterns among simian myeloid and enteric α-defensins were examined using the Gu99 and Gu2013 methods (Figure 2). Statistical analysis suggests that clusters of both simian myeloid α-defensins and simian enteric α-defensins have significantly diverged (Figure 2A). The pairwise coefficients of type I functional divergence (θ*_ij_*) are all significantly greater than 0 (*p*<0.05), except for the comparison between *DEFA9* and *DEFA6* (*p* = 0.07). The type I functional distance for each pair of clusters was estimated to measure whether one cluster has more shifted evolutionary rates than the other cluster following gene duplication [52]. The type I functional distance (*d*_F_) ranges from 0.78 to 1.40 for the simian myeloid α-defensins (with the functional branch length of *DEFA1*, *DEFA4* and *DEFA8* being 0.94, 0.32 and 0.46, respectively) and from 0.69 to 3.93 for the simian enteric α-defensins (with the functional branch length of *DEFA5*, *DEFA6* and *DEFA9* being 3.41, 0.53 and 0.17, respectively). Type I functional distances among enteric α-defensins are much higher than among myeloid ones, except for the distance between *DEFA9* and *DEFA6*. The enteric α-defensins have a higher level of functional divergence, which reflects a more challenging microbial environment in the gut. Critical amino acid sites responsible for the type I and type II functional divergences (hereafter referred to as type I and type II sites, respectively) were detected (Figure 2B and C). For simian myeloid α-defensin clusters, the type I sites of *DEFA1* are located in the mature peptide, whereas the type I sites of *DEFA8* and *DEFA4* are located in the signal-prosegment (Figure 2D). The different locations of the type I sites among simian myeloid α-defensin clusters suggest a specific altered functional constraint on the mature peptide of *DEFA1*. For simian enteric α-defensin clusters, the type I sites of *DEFA5* are located in the signal-prosegment, whereas the type I sites of *DEFA9* and *DEFA6* are primarily located in the mature peptide. This result indicates a specific altered functional constraint on the signal-prosegment region of *DEFA5*. Therefore, different clusters of α-defensins have undergone various functional constraints and have likely been specialized in their antimicrobial spectra. No site corresponding to the type I or type II functional divergence was detected between simian myeloid α-defensins and simian enteric α-defensins because of the high level of divergence within both clades.

**Figure 3:**
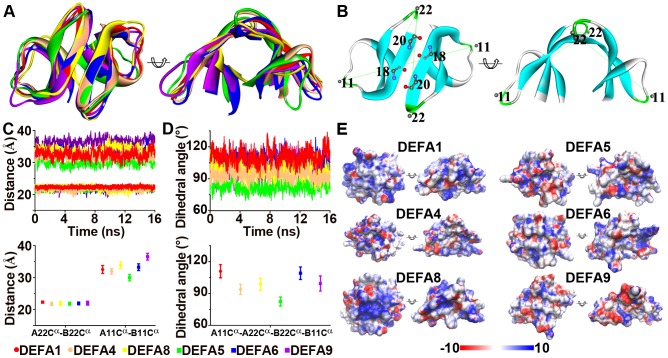
Structural flexibility and surface electrostatic potential for the simian myeloid and enteric α-defensin dimers. A: Superimposed α-defensin dimers for the myeloid DEFA1, DEFA4 and DEFA8 proteins as well as the enteric DEFA5, DEFA6 and DEFA9 proteins. B: Diagram illustrating the intermolecular hydrogen bonds, the two distances (A22C^α^-B22C^α^ and A11C^α^-B11C^α^) and the dihedral angle (A11C^α^-A22C^α^-B22C^α^-B11C^α^). C: Plots of the A22C^α^-B22C^α^ and A11C^α^-B11C^α^ distances during the 16 nanoseconds of molecular dynamics simulations (upper panel) and plots of the average A22C^α^-B22C^α^ and A11C^α^-B11C^α^ distances (lower panel). Similar A22C^α^-B22C^α^ distances indicate that the dimer structures are well maintained during simulation processes, whereas diverse A11C^α^-B11C^α^ distances reflect the flexibility of the dimers. Error bars represent standard deviations. D: Plots of the dihedral angle A11C^α^-A22C^α^-B22C^α^-B11C^α^. The variant dihedral angles among different clusters of myeloid and enteric α-defensins indicate different dimer topologies. E: Surface electrostatic potential generated using the smoothed trajectory from the last five frames of molecular dynamics simulations. The electrostatic potential (±10 kT/e) is colored red (−) or blue (+). The left and right views of each structure are the same as in panel A.

The dimerization and the cationic and amphipathic properties of the α-defensin structures are important for their antimicrobial activities [2], [33], [35]–[37]; hence, these characters of the representative structures from six clusters were analyzed. The α-defensin dimers were superimposed based on the backbone residues 18 and 20, which form four pairs of intermolecular hydrogen bonds (Figure 3A and B). These superimposed dimers include the myeloid α-defensins DEFA1 (human HNP1), DEFA4 (human HNP4) and DEFA8 (modeled rhesus macaque DEFA8) and the enteric α-defensins DEFA5 (human HD5), DEFA6 (human HD6) and DEFA9 (modeled marmoset DEFA9a). The β-sheets overlap well, whereas the two loops connecting β1-β2 strands and β2-β3 strands show structural variation. Residues 11 and 22 represent the most distal amino acids on the two flexible loops. Thus, the distances of A22C^α^-B22C^α^ and A11C^α^-B11C^α^, as well as the dihedral angle of A11C^α^-A22C^α^-B22C^α^-B11C^α^, were monitored in molecular dynamics simulations (see Materials and Methods) to assess their structural variation (Figure 3B). The distance of A22C^α^-B22C^α^ is almost equal among the six dimers (Figure 3C), suggesting that the dimers are not separated into monomers and remain stable during the 16-nanosecond molecular dynamics simulation. The distance of A11C^α^-B11C^α^ ranges from 30 Å to 37 Å, and the dihedral angle of A11C^α^-A22C^α^-B22C^α^-B11C^α^ ranges from 75° to 120° (Figure 3C and D), showing significant variations in these dimer structures and providing a structural foundation for their different antimicrobial activities. In addition to dimerization, the surface electrostatic potentials of simian enteric and myeloid α-defensins create distinct cationic and amphipathic patterns (Figure 3E), which likely affect their membrane depolarization and permeabilization abilities through electrostatic and hydrophobic interactions with microbial membranes.

### Sequence profiles and structural features of θ-defensins

**Figure 4:**
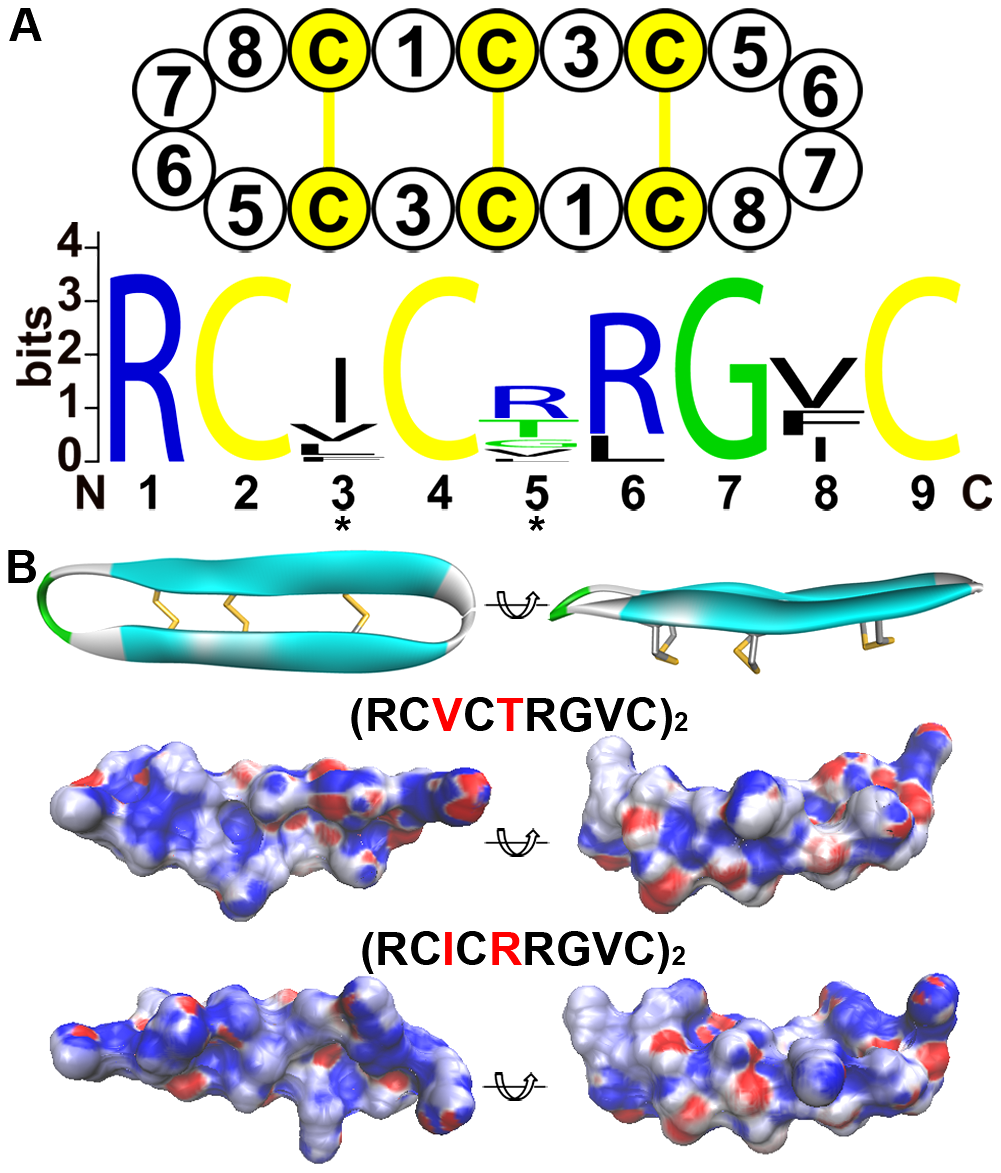
Sequence motifs and electrostatic features of primate cyclic θ-defensins. A: The θ-defensin octadecapeptide is formed by the head-to-tail ligation of two nonapeptides. The sequence logo for the nonapeptide is generated using all the primate θ-defensins. Sites 3I and 5R are under positive selection and are marked with asterisks. B: Surface electrostatic potential of the θ-defensins without or with the positively selected sites (3I and 5R).

The θ-defensins are cyclic octadecapeptides formed by the head-to-tail ligation of two nonapeptides that can be homodimeric or heterodimeric to increase their diversity [62], [65]. The nonapeptide motif of θ-defensins is presented as RC[IVLF]C[RTGVL][RL]G[VFI]C (Figure 4A), which shows both a conserved cysteine pattern and a conserved hydrophilic/hydrophobic pattern. The conserved cysteines at positions 2, 4 and 9 of the consensus motif form the tridisulfide ladder that has been proven to be important for structural stability but not for antimicrobial activity [66]. Positions 1, 5 and 6 are mostly cationic and hydrophilic arginine residues, whereas positions 3, 7 and 8 are mostly hydrophobic residues. The side chains of arginine residues and the tridisulfide ladder are on opposite sides of the cyclic backbone plane and thus form a polarized structure. The conserved hydrophilic/hydrophobic pattern generates the cationic and amphipathic properties, which might play a major role in the membrane depolarization and permeabilization of target cells or enveloped viruses. The cationic and amphipathic properties of θ-defensins, like α-defensins, have been reported to be important for antimicrobial activities by disrupting microbial membrane structures [2], [33].

Sites 3I and 5R in the nonapeptides (corresponding to 67I and 69R in the full sequence) were found to be under significant positive selection (Table 1). The surface electronic potentials for two types of θ-defensin homodimers, (RCICRRGVC)_2_ that is under positive selection and (RCVCTRGVC)_2_ that is not under positive selection (Figure 4B), were analyzed based on molecular dynamics simulations. The surface electronic potentials exhibit differences that may affect the polarization of θ-defensin, suggesting that the positively selected sites are related to the antimicrobial function of θ-defensin.

### Adaptive changes in the prosegment coevolving with the new protein fold of the mature peptide

**Figure 5:**
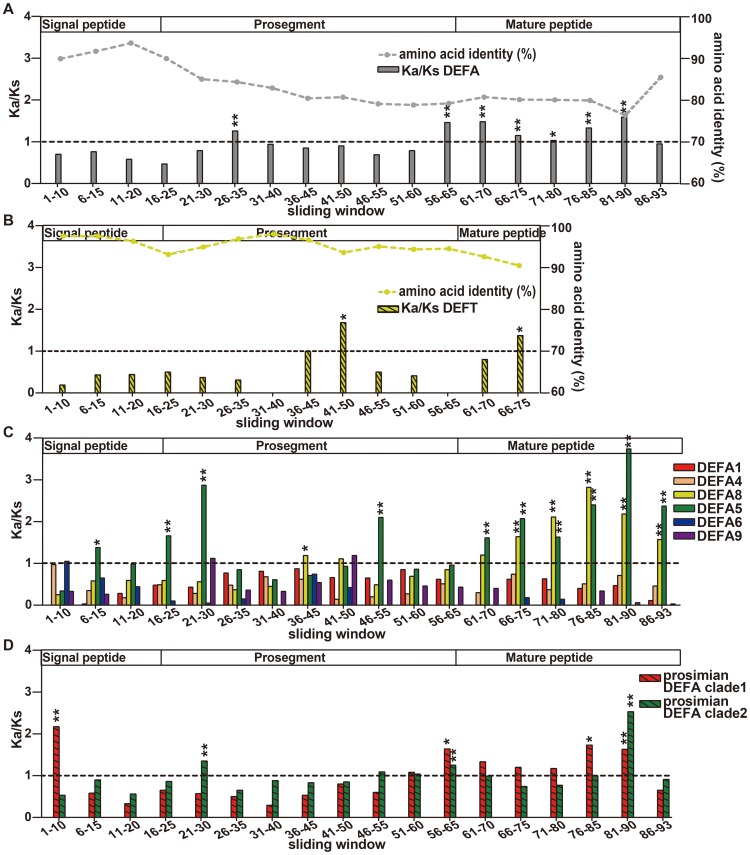
Sliding window analyses of the Ka/Ks for all *DEFA*, *DEFT* and separate *DEFA* clusters. A: Sliding window analysis of the amino acid identity and Ka/Ks for all *DEFA*. The sequence conservation decreases from the signal peptide and prosegment to the mature peptide. The selection pressures acting on different windows are different as indicated by the Ka/Ks ratios. Fragment 26–35 in the prosegment region, fragment 56–65 in the cleavage region and most fragments in the mature peptide are under positive selection (Ka/Ks>1). The significance of Ka/Ks>1 is tested by a bootstrap method and is indicated by * (P<0.05) or ** (P<0.01). B: Sliding window analysis of the amino acid identity and Ka/Ks for *DEFT*. The sequence conservation also decreases from the signal peptide and prosegment to the mature peptide. The selection pressure on different regions is also variable. Compared to the same region of all *DEFA* and that of *DEFA8*, fragment 41–50 in the prosegment of *DEFT* is under strong positive selection. C: Sliding window analysis of Ka/Ks for each simian *DEFA* cluster. Only the *DEFA5* and *DEFA8* clusters contain fragments that are under positive selection. D: Sliding window analysis of Ka/Ks for the two prosimian clades. The fragment 56–65 in the cleavage region is also under positive selection for both prosimian clades, consistent with that of all *DEFA*.

The θ-defensins are the youngest members of the defensin family. A premature stop codon mutation in the mature peptide gives birth to the θ-defensin, which contains a new protein fold that is different from that of α-defensins. The selection patterns along the length of *DEFA* and *DEFT* were examined to check whether there were any adaptive changes in the signal-prosegment region that are related to the new protein fold. For this purpose, a sliding window method for the amino acid identity and the Ka/Ks was used to analyze the selection pressures on different regions of all *DEFA*, *DEFT* and separate *DEFA* clusters (Figure 5, Table S3). Furthermore, bootstrap tests for the Ka/Ks were performed to assess the effect of potential biases from a few genes in the gene set and to test the significance of the difference between the average Ka/Ks ratio and 1.

The amino acid identities decrease from the signal peptide and prosegment to the mature peptide for both *DEFA* and *DEFT* (Figure 5A and B). The signal peptide is the most conserved region, whereas the mature peptide is highly divergent. This result also supported our rationale for inferring a phylogenetic tree using the more conserved signal-prosegment region (Figure 1A).

The Ka/Ks ratios are not homogeneous among the sliding windows for *DEFA*, *DEFT* and separate *DEFA* clusters, indicating that varying selection pressures act on different fragments. The signal peptides are tested to be under purifying selection (Ka/Ks<1) for both *DEFA* and *DEFT*. When the *DEFA* clusters are analyzed separately, only the *DEFA5* cluster (sites 6–15) and the prosimian *DEFA* clade 1 (sites 1–10) are detected with positive selection in the signal peptides. However, the mature peptides are under positive selection (Ka/Ks>1) for both *DEFA* and *DEFT*. For separate simian *DEFA* clusters, the mature peptides of *DEFA5* and *DEFA8* are under strong positive selection. The mature peptides of the prosimian *DEFA* clade 1 and clade 2 are also detected with positive selection. The positive selection acting on the mature peptides is likely due to the strong selection pressure through their direct interactions with variable microbes.

The prosegments of the *DEFA* and the *DEFT* are under different selection pressures. For *DEFA*, the fragment 56–65 in the prosegment is under positive selection (Ka/Ks = 1.46, bootstrap test Ka/Ks significantly >1, P<0.01) (Figure 5A), whereas the same fragment from separate simian *DEFA* clusters (Figure 5C) is not detected with positive selection. The fragment 56–65 is a crucial region that harbors several posttranslational cleavage sites [31], [67], [68]. The positive selection acting on this cleavage region of *DEFA* may be explained by the diverse splicing mechanisms among different *DEFA* clusters, which are conserved within each cluster. The prosimian clade 1 and clade 2 are also detected to be under positive selection in the cleavage region, indicating that both clades contain divergent α-defensins.

**Figure 6:**
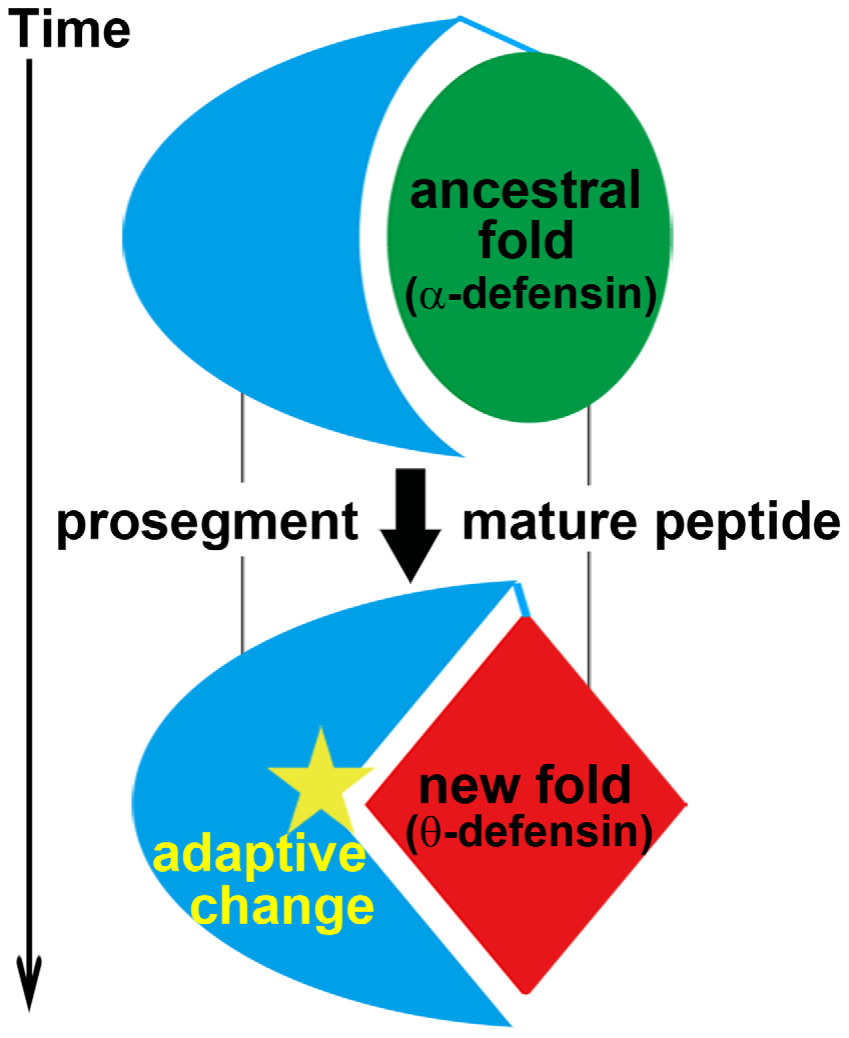
Coevolution of the prosegment with the new protein fold of the mature peptide. The ancestral protein fold refers to the α-defensin mature peptide, and the new protein fold refers to the θ-defensin mature peptide. Because the prosegment is important for the correct folding of the mature peptide, the prosegment coevolves with the mature peptide through adaptive changes.

For *DEFT*, the fragment 41–50 in the prosegment is under strong positive selection (Ka/Ks = 1.68, Ka/Ks significantly >1, P<0.05), compared to the corresponding fragment of *DEFA* (Ka/Ks significantly <1, P<0.01) and the closely related *DEFA8* (Ka/Ks = 1.11, Ka/Ks not significantly >1, P = 0.16). However, the prosegment of *DEFA* is detected with positive selection in another region, namely, the fragment 26–35 (Ka/Ks = 1.26, Ka/Ks significantly >1, P<0.01). The distinct positive selection in the prosegments of *DEFA* and *DEFT* indicates a specific change in the prosegment of *DEFT*. Because the prosegment is important for the correct folding of defensins [29], it is most likely that the prosegment of θ-defensins has undergone adaptive changes that are related to the new protein fold. The diagram in Figure 6 presents our proposed hypothesis for this evolutionary pattern and illustrates the adaptive evolution of the prosegment along with the new protein fold of the mature peptide.

### Genetic hitchhiking of the pseudogene *DEFTP* and loss of functional θ-defensin in humans and chimpanzees

**Figure 7:**
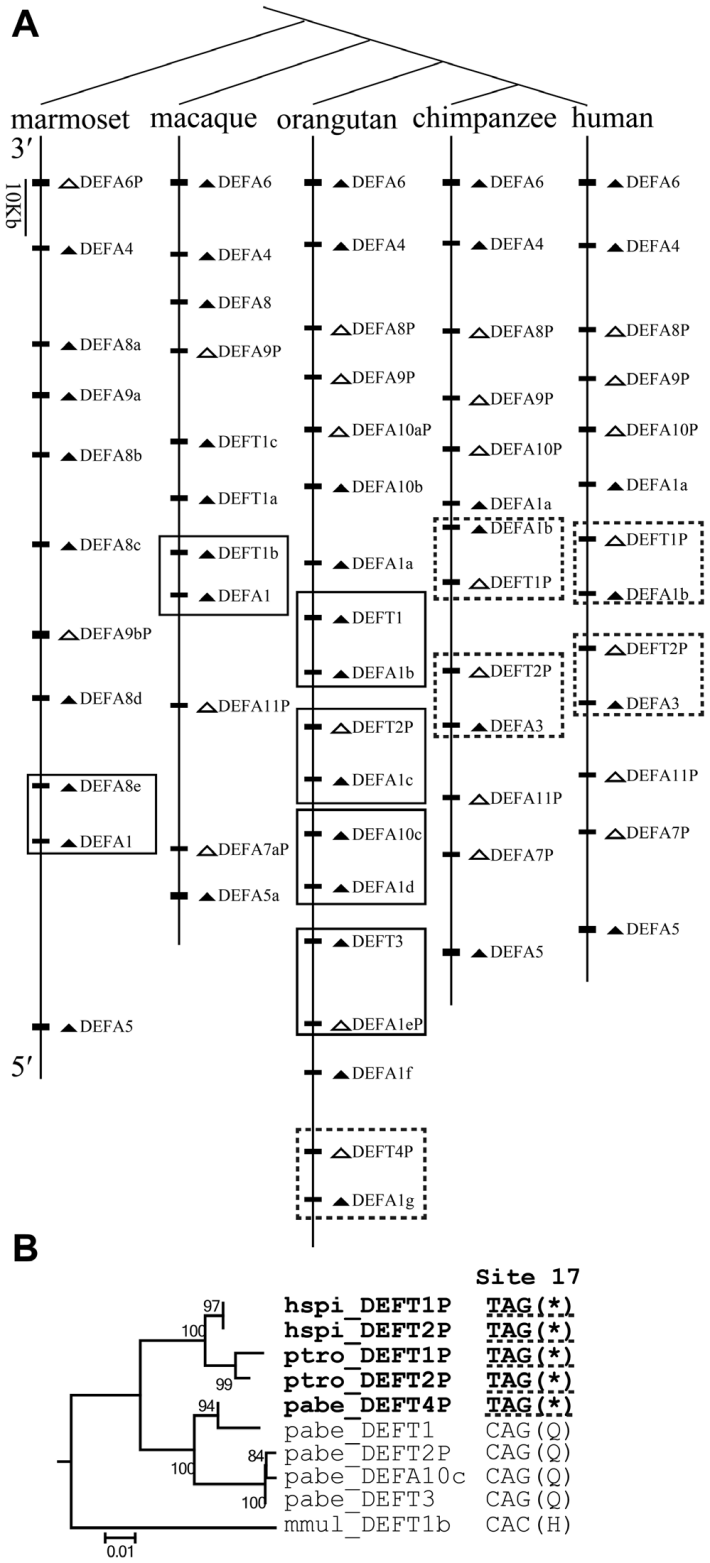
Hitchhiking of *DEFT/DEFTP* during the birth-and-death evolution of the primate *DEFA*/*DEFT* multigene family. A: Comparative synteny map of the *DEFA*/*DEFT* gene loci in humans, chimpanzees, orangutans, macaques and marmosets. The boxes highlight the two genes that are linked and duplicate together, including a driver from the *DEFA1* cluster and a hitchhiker from the *DEFT/DEFA10* cluster. Arrowheads indicate transcriptional orientation. Pseudogenes are in white, and functional genes are in black. The dashed-line box includes the pseudogene *DEFTP* containing the nonsense mutation at site 17. B: The NJ tree based on the introns of the boxed *DEFT/DEFTP* from humans (*hspi*), chimpanzees (*ptro*), orangutans (*pabe*) and macaques (*mmul*). The nonsense mutations (*) at codon site 17 of the pseudogenes *hspi_DEFT1P*, *hspi_DEFT2P ptro_DEFT1P*, *ptro_DEFT2P* and *pabe_DEFT4P* are underlined with dashed lines, suggesting that these *DEFTP* pseudogenes are derived from a common ancestor.

To understand the evolutionary dynamics of the α-/θ-defensin multigene family, the comparative synteny map from five simian species, including humans, chimpanzees, orangutans, macaques and marmosets, was analyzed and was combined with the phylogenetic results. The primate *DEFA/DEFT* genes originate from tandem duplication and are located in the regions homologous to the human chromosome 8p23 (Figure 7A). Conserved synteny relationships are observed among the five simian species. The *DEFA5* orthologs are located at the 5′ terminus, and the *DEFA9*, *DEFA8*, *DEFA4* and *DEFA6* orthologs are located at the 3′ terminus in a 5′ to 3′ order. The simian myeloid and enteric *DEFA* genes, which are clustered into distinct clades in both phylogenetic trees (Figure 1), are not located as two clearly separated blocks in the synteny map. Loci of the genes from the two major clades (simian enteric and myeloid defensins) are intermingled, implying that duplication of a segment containing two or more genes has occurred. Orthologs from some species have mutated into pseudogenes, which include *DEFA6P* in the marmoset; *DEFA9P* in the macaque, orangutan, chimpanzee and human; and *DEFA8P* in the orangutan, chimpanzee and human. In the phylogenetic trees, genes from the same species group together in each simian cluster (Figure 1), indicating species-specific duplication, which is a common phenomenon during the birth-and-death evolution of multigene families. In the synteny map (Figure 7A), species-specific duplication is not homogeneous across this region, with the middle section having more species-specific duplications. Based on the current versions of assembled genomes, the marmoset has multiple duplications of *DEFA8* and *DEFA9*, and the macaque has multiple duplications of *DEFT*. In the orangutan, chimpanzee and human, there are several species-specific duplications of *DEFA1* and *DEFA10/DEFT*.

A nonsense mutation at codon 17 of *DEFT* results in its loss of function in orangutans, chimpanzees and humans (Figure 7B), and these *DEFTP* pseudogenes contain the *hspi_DEFT1P*, *hspi_DEFT2P*, *ptro_DEFT1P*, *ptro_DEFT2P* and *pabe_DEFT4P* (excluding the *pabe_DEFT2P* pseudogene which is generated by frame-shift mutations). In humans, chimpanzees and gorillas, the *DEFT* has been lost, and only *DEFTP* pseudogenes have been maintained. Our results demonstrate that θ-defensin had a conserved structural motif and was under positive selection. Therefore, the driving force for the expansion of the pseudogene *DEFTP* and for the loss of functionally important θ-defensins in humans and chimpanzees were investigated. A less-is-hitchhiking hypothesis was proposed based on the synteny map and phylogenetic relationships. The synteny map suggests that the loci of the *DEFA1* and the *DEFT/DEFA10* are linked and have been duplicated together in orangutans, chimpanzees and humans (Figure 7A). The genes of the *DEFA1* cluster are all functional and are under positive selection; thus, we infer that each box in the synteny map contains a driver from the *DEFA1* cluster and a hitchhiker from the *DEFT/DEFA10* cluster. The orangutan has multiple copies of functional *DEFT* as well as the pseudogene *pabe_DEFT4P* containing the nonsense mutation at site 17 (Figure 7A and B). This result suggests that, in the ancestor of the orangutan, chimpanzee and human, the selection pressure on the multiple-copied *DEFT* was relaxed and the pseudogene *DEFTP* (containing the nonsense mutation at site 17) emerged. Both *DEFT* and *DEFTP* are linked to *DEFA1* and can expand, but only *DEFTP* (containing the nonsense mutation at site 17) is maintained, whereas *DEFT* is lost in the chimpanzees and humans (Figure 7A). The retention of *DEFTP* and loss of *DEFT* might be independent of their own fitness and depends on the fate of the driver. The *pabe_DEFT4P* is linked to the *pabe_DEFA1g*, which harbors a significantly positively selected site whose expansion might be advantageous: 74I, adjacent to the third cysteine. This 74I is not present in other *pabe_DEFA1* (*a*, *b*, *c*, *d and f*) sequences (Table 1, Figure S1). Therefore, the *DEFTP* is linked to a *DEFA1*, whose expansion might be advantageous.

**Figure 8:**
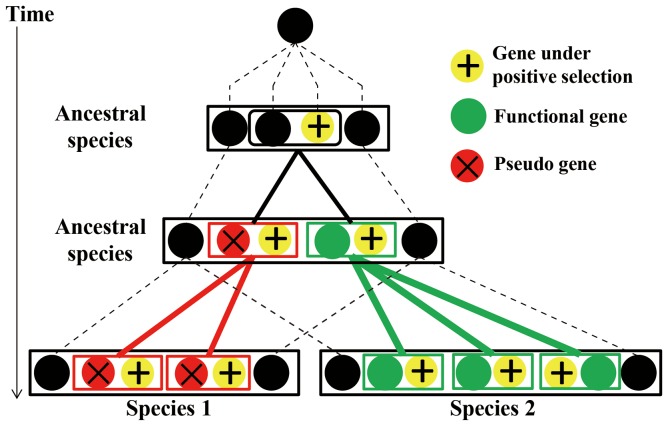
The less-is-hitchhiking hypothesis during the birth-and-death evolution of multigene families. With the expansion of the gene (driver) under positive selection, the adjacent linked gene (hitchhiker) is duplicated in the process of segmental duplication. Pseudogenization of the hitchhiker occurs in an ancestral species following the duplication process. During the process of birth-and-death evolution, the hitchhiker that gains more copies can be either the pseudogene or the functional gene with the other one being lost, and the gain or loss of the hitchhiker is determined by the fitness of its adjacent driver. The expansion of the pseudogene and loss of the functional gene scenario is defined as the less-is-hitchhiking hypothesis.

The less-is-hitchhiking hypothesis depicts the evolutionary process of the expansion of the pseudogene *DEFTP* and the loss of functional θ-defensin in the humans and chimpanzees (Figure 8). With the expansion of the gene (driver) under positive selection, the adjacent linked gene (hitchhiker) is duplicated in the process of segmental duplication. The pseudogenization of the hitchhiker occurs in an ancestral species following the duplication process. During the birth-and-death evolution, the hitchhiker that gains more copies may be either the pseudogene or the functional gene, which is determined by the fate of its adjacent driver. The former scenario is the less-is-hitchhiking hypothesis. The less-is-more, less-is-less and less-is-nothing hypotheses depend on the phenotypic change of the loss-of-function variant, which can be advantageous, deleterious or tolerated, respectively. However, the less-is-hitchhiking hypothesis provides another explanation for the driving force of the loss-of-function variant expansion that does not involve phenotypic consequences. The fate of the loss-of-function hitchhiker is determined by the surrounding genomic environment of a driver gene (i.e., an adjacent gene under positive selection). The loss-of-function hitchhiker can be advantageous, deleterious or tolerated.

## Discussion

In this study, we collected the primate *DEFA/DEFT* gene repertoires and performed extensive analyses of the primate α-/θ-defensin multigene family. Through systematic phylogenetic analyses, a detailed classification and nomenclature of the primate α-/θ-defensins was provided. The classification of simian α-/θ-defensins based on phylogenetic trees is related to their expression patterns in myeloid and enteric tissues. Further phylogenetic classification of simian α-defensins into six functional gene clusters corresponds to their functional divergence. In a previous study, this multigene family was classified into three classes based on neighbor-joining (NJ) and maximum parsimony (MP) trees using genes from simians that included human, chimpanzee, orangutan, macaque and marmoset sequences as well as one mouse α-defensin sequence as the outgroup [42]. A recent study focused on the phylogenetic relationships of the α-, β- and θ-defensins using *DEFA/DEFT* sequences from 10 primate species and two human *DEFB* sequences as the outgroup based on the NJ method [69]. In our study, we identified 144 primate *DEFA/DEFT* sequences from 16 species of simians and prosimians as well as 10 *DEFA* sequences from two treeshrews, which are closely related to primates. We used sequence alignments of both the more conserved signal-prosegment region and the entire coding region to more precisely reconstruct α-/θ-defensin orthologous relationships and to uncover their potential functional divergence.

The primate *DEFA/DEFT* sequences that are used for this study were collected from the public database, which may contain errors or uncertainty of the sequencing and assembly. One of such ambiguous sequences might be the *pabe_DEFA10c* sequence, which is highly similar to the *pabe_DEFT* sequences and is phylogenetically far from the *DEFA10* cluster (Figure 1A and S8). This sequence is named *DEFA10* only because site 77 is not a premature stop codon. Thus, *pabe_DEFA10c* was excluded in the phylogenetic analysis based on the introns from *DEFA8/DEFA10/DEFT* (Figure S9). For the analysis of positive selection using PAML, sequencing and assembly errors may cause bias. Our results of PAML analysis for separate clusters show that most of the positively selected sites are located in the mature peptide, similar to the results of a previous study that analyzed all the *DEFA* sequences together [42]. For the sliding window analysis of Ka/Ks, we used a bootstrap method to eliminate the effect of potential bias from the sequencing and assembly error in a few genes. For the synteny analysis, there are sequences (*cjac_DEFA9cU* and *cjac_DEFA9dU*) that are not mapped onto the chromosomes of the marmoset. In macaques, several sequences (*mmul_7bUP*, *mmul_DEFA5bU*, *mmul_DEFA5cU*, *mmul_DEFA5dU* and *mmul_DEFA5eU*) are mapped onto a chromosome region that is quite far from the defensin gene locus presented in the synteny map. The human, chimpanzee, orangutan, macaque and marmoset genomes were selected for the synteny analysis because of their more complete assemblies in the genomic region harboring the *DEFA1* and *DEFA8/DEFA10/DEFT* gene cluster, whereas the gorilla and gibbon genomes were not used due to the poor assembly in this region.

The functional shifting of simian α-defensins, which is implied by their sequence divergence, structural variation and selection pattern differences among the six functional gene clusters, most likely corresponds to differentiated antimicrobial activity. However, orthologs within the same gene cluster from different species are still under functional constraints, most likely due to their similar antimicrobial mechanisms and challenges from similar types of pathogens in closely related species. Based on these results, we speculate that these defensin clusters constitute a wide range of antimicrobial spectra and that each cluster occupies one part of the spectra. Within each cluster, there is species-specific cluster duplication or pseudogenization following the species diversifications of simians, which implies that the antimicrobial spectra are continuously changing. At the same time, deconstructing the undocumented antimicrobial spectra of α-/θ-defensins is not feasible, even for the most closely related species, because of the countless types of pathogens and the frequent duplication and divergence of defensins. Many experimental studies have shown that members of this family have different antimicrobial activities and are effective against different groups of microbes. For example, the antibacterial activity and specificity of the six human α-defensins against Gram-positive and Gram-negative bacteria are different [70]. For human myeloid defensins, HNP1-3 defensins are of greater potencies than HNP4 against Gram-positive bacteria such as *S. aureus*, whereas HNP4 is of greater potency against Gram-negative bacteria such as *E. coli* and *E. aerogenes*. For human enteric defensins, HD5 is effective against both Gram-negative and Gram-positive bacteria, whereas HD6 shows significantly lower antimicrobial activity against Gram-negative and Gram-positive bacteria than HD5. The myeloid α-/θ-defensins and the enteric HD5 have also been reported to have inhibitory potential against enveloped and non-enveloped viruses, such as the herpes simplex virus [71], the human immunodeficiency virus [72], [73], papillomaviruses [74], adenoviruses [75], polyomaviruses [76], the SARS coronavirus [77], influenza viruses [78], [79] and others. In addition to its role in host defense, HD5 also has a homeostatic role in establishing and maintaining the intestinal microbiota [80]. Overall, these experimental studies indicate that the variable antimicrobial abilities of mammalian α-/θ-defensins compose an antimicrobial spectrum. Defensins with continuously shifting antimicrobial activity, together with other versatile antimicrobial factors, evolve to act as the first line of defense of the innate immune system and play important roles in the early host defense. Because of the therapeutic potential of antimicrobial peptides, synthetic defensins are being developed as peptide drugs, such as the fusion inhibitors. Exploring the natural defensin repertories, especially in species closely related to humans, will help us understand the stability and plasticity of α-/θ-defensins and aid in designing potent peptide drugs.

The sliding window analysis of *DEFT* (Figure 5B) suggests that fragment 41–50 in the prosegment and fragment 66–75 in the mature peptide are under positive selection. These two fragments contain the positively selected sites detected by PAML (50D, 67I and 69R, corresponding to the sliding window sites 49, 66 and 68, respectively). Other amino acid changes that may contribute to the high Ka/Ks value are 41G/E, 45S/A and 48R/W in fragment 41–50 as well as 69R/L, 71V/I/F and 73R/Q in fragment 66–75 (Figure S1). To be spliced into an 18-residue mature θ-defensin, the three C-terminal residues must be removed during the maturation process [18]. The three C-terminal residues were not found to be under positive selection using the PAML software. However, the three C-terminal residues of θ-defensin show a pattern of RLL or QLL that is quite different from the α-defensins (Figure S1), which might be related to their involvement in the maturation process of θ-defensins.

Multigene families play important roles in the immune system, the sensory system, development and other processes. These families can express gene products at high levels, such as highly conserved histones and nuclear ribosomal RNAs, and produce proteins with diverse functional spectra, such as α-defensins, major histocompatibility complex proteins, immunoglobulins, chemoreceptors and olfactory receptors. The evolutionary processes of multigene families have been explained by birth-and-death evolutionary models [43]. Frequent gene conversion, interlocus recombination, gene duplication and pseudogenization are involved in the evolutionary processes of multigene families. Strong purifying selection or positive selection can also act on multigene families to conserve gene function or give rise to new adaptive phenotypes. The expansion of genes under selection through segmental duplications may impact the fate of the adjacent genes linked with them, which can also affect the evolution of multigene families. Our less-is-hitchhiking hypothesis depicts this phenomenon that the retention of pseudogenes and the loss of functional gene are determined by the fate of the adjacent gene during the birth-and-death evolutionary process. We believe that the expansion of the *DEFTP* pseudogene and the loss of functional θ-defensins in humans and chimpanzees is a representative case for the less-is-hitchhiking hypothesis based on the following criteria. First, the driver and the hitchhiker belong to phylogenetically separate clusters. Second, the driver and the hitchhiker are genetically linked and duplicate together on the chromosome. Third, positive selection acts on the functional drivers. Lastly, the pseudogene hitchhiker expands and the functional hitchhiker has been lost, as determined by the fitness of the driver.

Recently, it has been demonstrated that genetic hitchhiking is pervasive and the mutational cohort that includes both the driver and the hitchhikers drives adaptation [81]. Although these hitchhikers mostly refer to point mutations, there are few reports of an entire gene being a hitchhiker, with one exception reported for the yellow monkey flower (*Mimulus guttatus*), in which a copper tolerance locus under selection and its tightly linked hybrid incompatibility locus spread to fixation in a copper mine population by genetic hitchhiking [82], [83]. Future research on the evolution of genetic hitchhiking involving two or more closely linked genes from both case studies and whole-genome comparisons will help to uncover the adaptation of complex traits from linked genes and to understand the genetic and evolutionary basis of certain disease-related traits during the hitchhiking processes.

## Supporting Information

Figure S1 **Multiple sequence alignment of the primate α-/θ-defensins.** Most of the protein sequences are computationally translated from genome sequences rather than being demonstrated to be produced in vivo. The mature peptide of α-defensins has a motif with six conserved cysteine residues, represented as C-x-C-x(3,4)-C-x(9)-C-x(6,9)-C. A nonsense mutation at position 77 (Q77*) generates a θ-defensin precursor with three conserved cysteines. The mature peptide of θ-defensins has a nonapeptide motif with three conserved cysteine residues, represented as x-C-x-C-x(4)-C, with the exception of the Eastern black-and-white colobus θ-defensin (*cgue_DEFT*), which has the motif x-C-x-C-x(8)-C. The nonsense mutation at position 17 (Q17*) results in the loss of the downstream protein-coding ability of the θ-defensins. The initial methionines (M) and conserved cysteines (C) in the alignment are highlighted in blue. The translational ends (*) of the θ-defensin are highlighted in magenta. The blocks highlight the sliding windows of fragments 41–50 and 65–75 of *DEFT*, which are detected with Ka/Ks >1 (P<0.05) in Figure 5. The question marks indicate ambiguous amino acids because of the single nucleotide polymorphisms from the genome sequences. The genus and species for each abbreviation can be found in Table S1.
(PDF)

Figure S2 **Phylogenetic trees of primate and treeshrew DEFA/DEFT genes based on the signal-prosegment region.** The trees are built using the (A) Bayesian inference (BI), (B) neighbor-joining (NJ) and (C) maximum likelihood (ML) methods. The BI tree is labeled with posterior probabilities. The NJ and ML trees are labeled with bootstrap support values. All three trees are drawn to scale, with branch lengths proportional to the estimated evolutionary distances. “P” in node labels denotes a pseudogene.
(PDF)

Figure S3 **Phylogenetic tree of primate and treeshrew DEFA/DEFT genes based on the signal-prosegment region.** The BI tree is selected as the background tree. The major clades or clusters having similar topologies from all three tree-building methods (BI, NJ and ML) are combined and labeled with the BI posterior probabilities and the bootstrap support values from the NJ and ML analyses.
(PDF)

Figure S4 **Phylogenetic trees of primate and treeshrew** ***DEFA*** **/** ***DEFT*** **genes based on the entire coding region.** The trees are built using the BI (A), NJ (B) and ML (C) methods. The BI tree is labeled with posterior probabilities. The NJ and ML trees are labeled with bootstrap support values. All three trees are drawn to scale, with branch lengths proportional to the estimated evolutionary distances.
(PDF)

Figure S5 **Phylogenetic tree of primate and treeshrew** ***DEFA*** **/** ***DEFT*** **genes based on the entire coding region.** The BI tree is selected as the background tree. The major clades or clusters having similar topologies from all three tree-building methods (BI, NJ and ML) are combined and labeled with the BI posterior probabilities and the bootstrap support values from the NJ and ML analyses.
(PDF)

Figure S6 **Phylogenetic incongruence is caused by the long-branch attraction of homogeneity sites.** The homogeneity sites under convergent or parallel evolution in the mature peptide (62, 63, 64, 65, 68, 71, 77, 81, 88, 89, 92 and 93) are highlighted in different colors. These homogeneity sites can cause phylogenetic incongruence between the trees constructed using the entire coding region versus the signal-prosegment region. The phylogenetic tree on the left is inferred based on the amino acid sequences of the entire coding region using NJ method without removing the homogeneity sites, whereas the tree on the right is built using NJ method after removing the homogeneity sites. When the long-branch attraction effect is eliminated, the sequences of the prosimian *DEFA* clade 2 group together.
(PDF)

Figure S7 **The different phylogenetic relationships of** ***DEFA9/DEFA11*** **are determined by different parts of the gene.** A: Different patterns of sequence similarity are observed before and after a clear boundary in the *DEFA9/DEFA11* genes, namely part I and part II. Phylogenetic trees are separately inferred from part I (the tree on the left) and part II (the tree on the right), using the *DEFA6* cluster as the outgroup. The combined tree based on part I shows that *DEFA11* is duplicated from *DEFA9* after the split of New World and Old World monkeys. Whereas, in the combined tree of part II, *DEFA11* is the outgroup of all *DEFA9* sequences. The BI tree is computed using the GTR + G + I (4 categories) model. The NJ tree is computed using the K2 + G (shape parameter  = 1.8 for part I and 3.3 for part II) model. The ML tree is computed using the K2 + G (4 categories) model. B: The BI/NJ/ML trees with branch lengths proportional to the estimated distances inferred from part I. C: The BI/NJ/ML trees inferred from part II.
(PDF)

Figure S8 **The different phylogenetic positions of hominoid** ***DEFA8*** **are determined by different parts of the gene.** A: Different patterns of sequence similarity are also observed before and after a clear boundary in the *DEFA8/DEFA10/DEFT* genes, which is different from that of *DEFA9/DEFA11* genes. Similarly, phylogenetic trees are separately inferred from part I (the tree on the left) and part II (the tree on the right), using the *DEFA1* cluster as the outgroup. In the combined tree inferred from part I, the *DEFA8* from Old World monkeys and hominoids clusters together with *DEFA10/DEFT*. Whereas in the tree inferred from part II, the *DEFA8* from Old World monkeys and hominoids is the outgroup in the *DEFA8/DEFA10/DEFT* cluster. The BI tree is computed using the GTR + G + I (4 categories) model. The NJ tree is computed using the K2 + G (shape parameter  = 1.5 for part I and 2.0 for part II) model. The ML tree is computed using the K2 + G (4 categories) model. The sequence *pabe_DEFA10c* is clustered with *DEFT*, likely resulting from sequence assembly error. Thus, the sequence *pabe_DEFA10c* was excluded in following analysis in Figure S9. B: The BI/NJ/ML trees with branch lengths proportional to the estimated distances inferred from part I. C: The BI/NJ/ML trees inferred from part II.
(PDF)

Figure S9 **Phylogenetic trees constructed using BI (A), NJ (B) and ML (C) methods based on the introns of** ***DEFA8/DEFA10/DEFT*** **.** *DEFT* is found in Old World monkeys and hominoids, whereas *DEFA10* is found in hominoids only. The *DEFA10* and *DEFT* are clustered into independent groups and are both derived from the duplication of *DEFA8* before the divergence of Old World monkeys and hominoids. But the *DEFA10* is lost in the ancestor of Old World monkeys. The BI tree is computed using the GTR + G + I (4 categories) model. The NJ tree is computed using the K2 + G (shape parameter  = 1.8) model. The ML tree is computed using the K2 + G (4 categories) model.
(PDF)

Table S1 **The repertoires of α-/θ-defensin (** ***DEFA/DEFT*** **) genes from primates and treeshrews.**
(PDF)

Table S2 **Log-likelihood values and parameter estimates for primate DEFA/DEFT genes under site models.** Notes: ω = dN/dS; p, number of free parameters; positively selected sites with significance at the 99% level are bold, whereas the remaining sites have significance at the 95% level. The likelihood ratio tests are analyzed by comparing the following pairs of models: M1a-M2a, M7-M8 and M8a-M8.
(PDF)

Table S3 **The average values of Ka, Ks and Ka/Ks from the sliding window analyses for the primate DEFA/DEFT genes and the P values from the bootstrap test.** Notes: avg. Ka: average pairwise Ka value; avg. Ks: average pairwise Ks value; avg. Ka/Ks: average pairwise Ka/Ks ratio; Bold number: Ka/Ks significantly >1 (P<0.05); NA: Not available.
(PDF)
