# Supplementary figures and images for: Molecular Evolution of the Primate α-/θ-Defensin Multigene Family
Source: PLoS One. 2014 May 12;9(5):e97425. doi: 10.1371/journal.pone.0097425 (PMC4018336; doi:10.1371/journal.pone.0097425)

**A**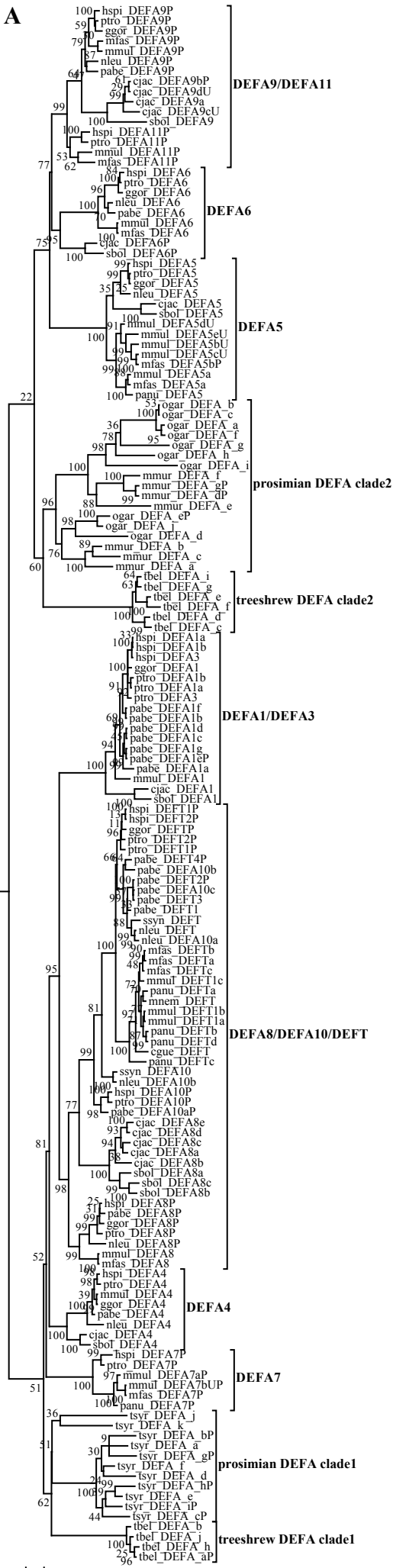**BI****B**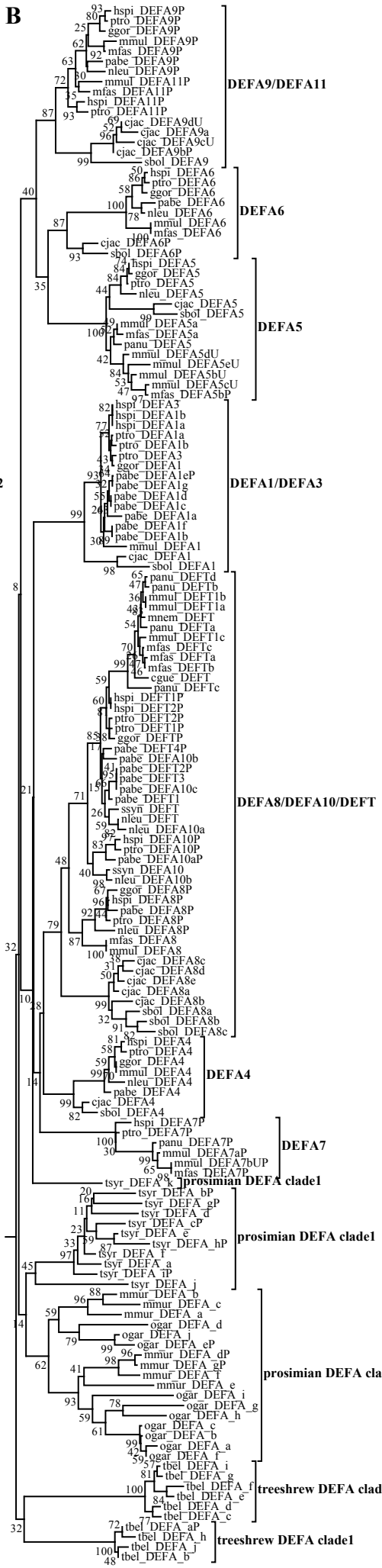**NJ****C**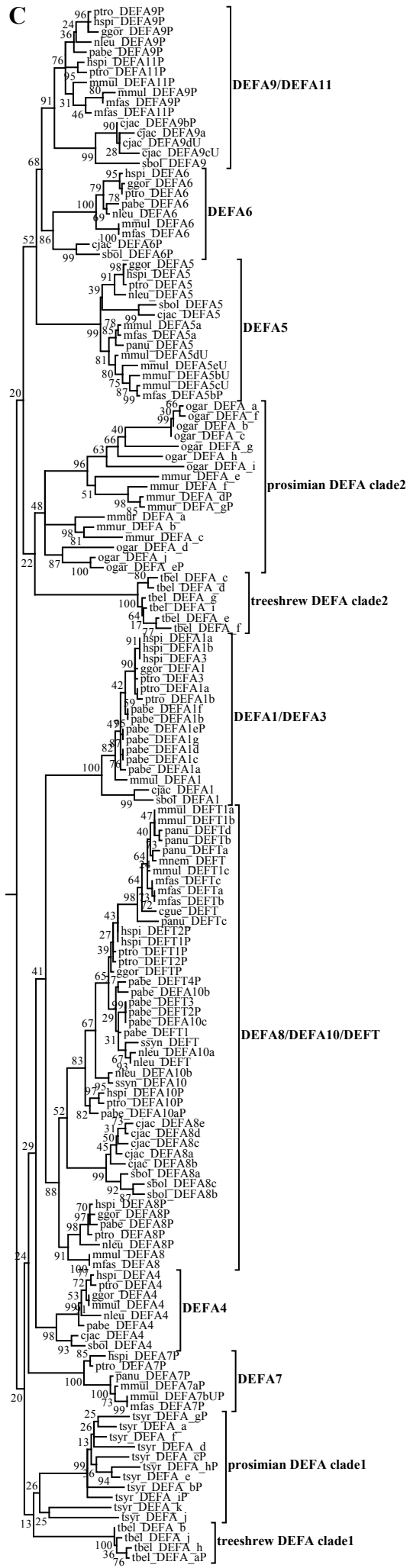**ML**

Supplement: Figure S2 — Phylogenetic trees of primate and treeshrew DEFA/DEFT genes based on the signal-prosegment region. The trees are built using the (A) Bayesian inference (BI), (B) neighbor-joining (NJ) and (C) maximum likelihood (ML) methods. The BI tree is labeled with posterior probabilities. The NJ and ML trees are labeled with bootstrap support values. All three trees are drawn to scale, with branch lengths proportional to the estimated evolutionary distances. “P” in node labels denotes a pseudogene. (PDF) [file pone.0097425.s002.pdf]

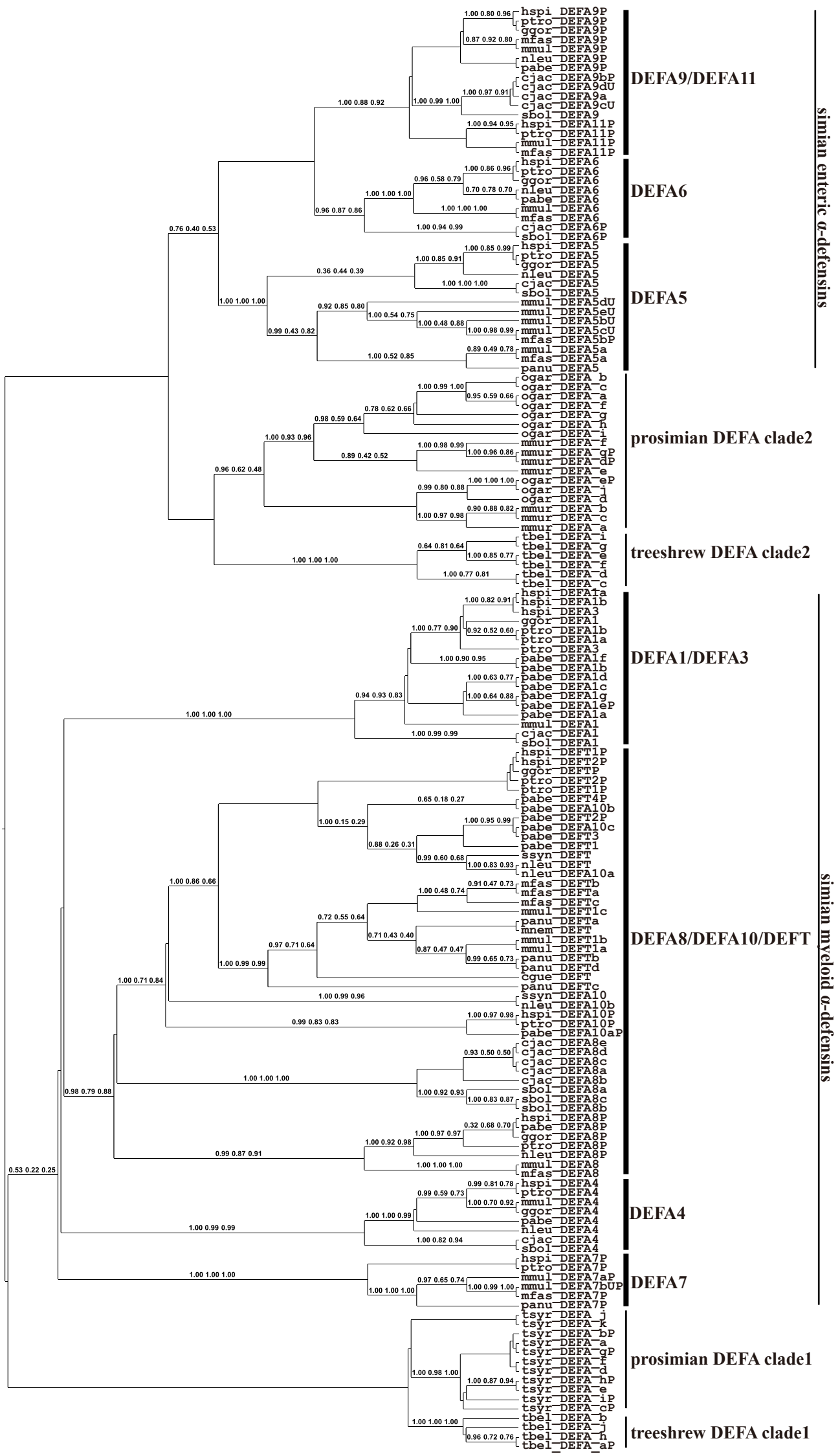

Supplement: Figure S3 — Phylogenetic tree of primate and treeshrew DEFA/DEFT genes based on the signal-prosegment region. The BI tree is selected as the background tree. The major clades or clusters having similar topologies from all three tree-building methods (BI, NJ and ML) are combined and labeled with the BI posterior probabilities and the bootstrap support values from the NJ and ML analyses. (PDF) [file pone.0097425.s003.pdf]

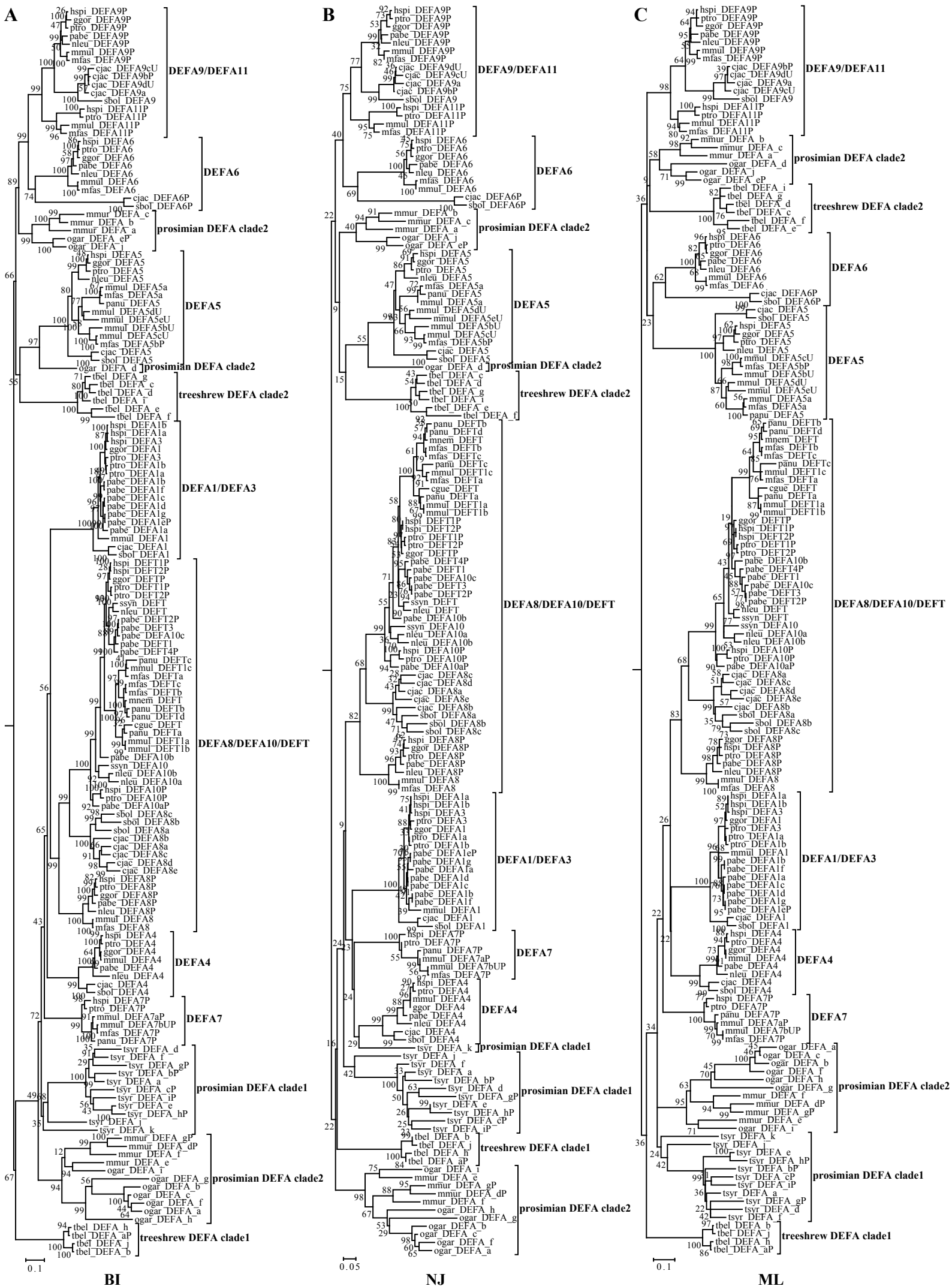

Supplement: Figure S4 — Phylogenetic trees of primate and treeshrew DEFA / DEFT genes based on the entire coding region. The trees are built using the BI (A), NJ (B) and ML (C) methods. The BI tree is labeled with posterior probabilities. The NJ and ML trees are labeled with bootstrap support values. All three trees are drawn to scale, with branch lengths proportional to the estimated evolutionary distances. (PDF) [file pone.0097425.s004.pdf]

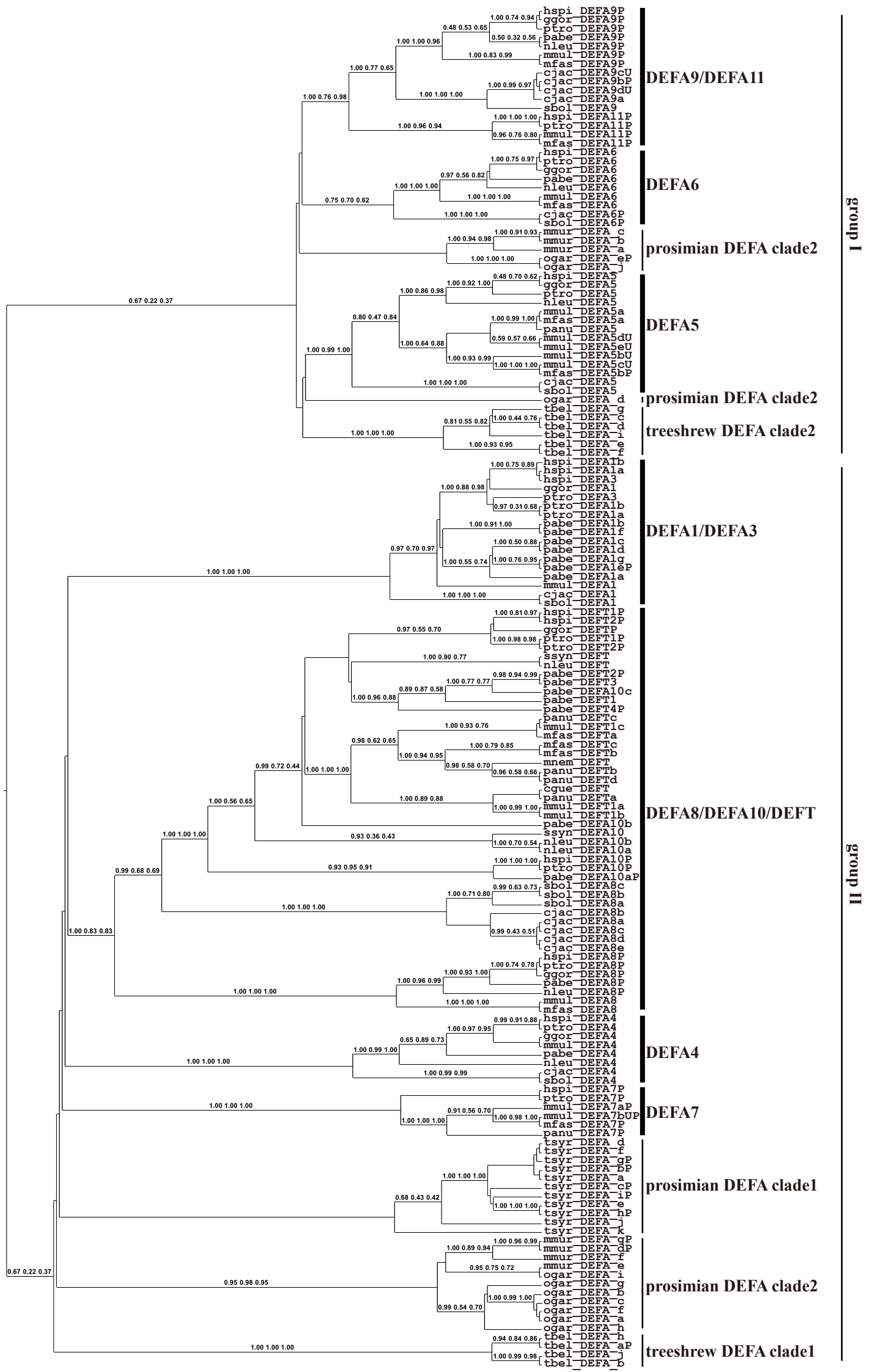

Supplement: Figure S5 — Phylogenetic tree of primate and treeshrew DEFA / DEFT genes based on the entire coding region. The BI tree is selected as the background tree. The major clades or clusters having similar topologies from all three tree-building methods (BI, NJ and ML) are combined and labeled with the BI posterior probabilities and the bootstrap support values from the NJ and ML analyses. (PDF) [file pone.0097425.s005.pdf]

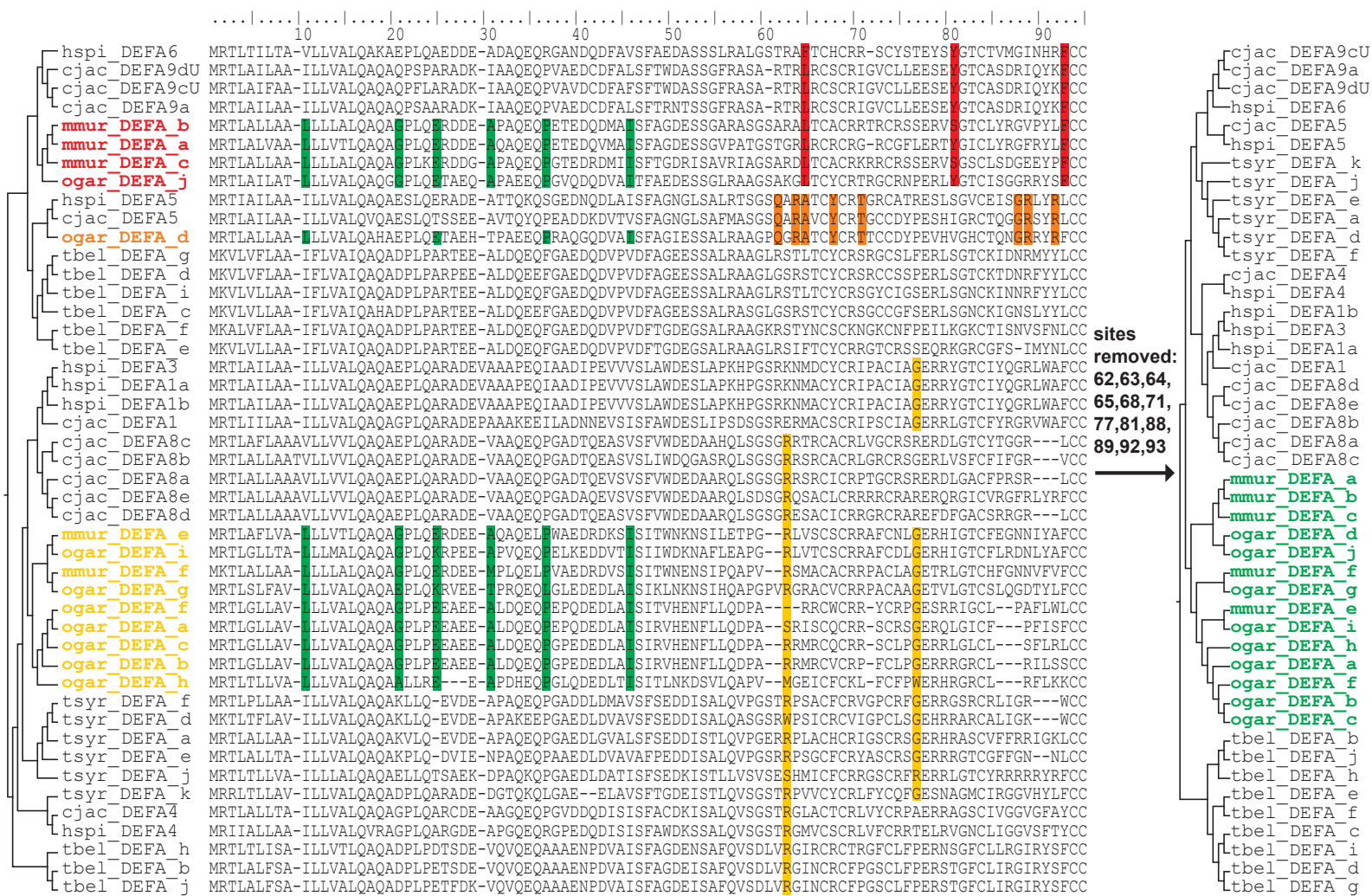

Supplement: Figure S6 — Phylogenetic incongruence is caused by the long-branch attraction of homogeneity sites. The homogeneity sites under convergent or parallel evolution in the mature peptide (62, 63, 64, 65, 68, 71, 77, 81, 88, 89, 92 and 93) are highlighted in different colors. These homogeneity sites can cause phylogenetic incongruence between the trees constructed using the entire coding region versus the signal-prosegment region. The phylogenetic tree on the left is inferred based on the amino acid sequences of the entire coding region using NJ method without removing the homogeneity sites, whereas the tree on the right is built using NJ method after removing the homogeneity sites. When the long-branch attraction effect is eliminated, the sequences of the prosimian DEFA clade 2 group together. (PDF) [file pone.0097425.s006.pdf]

**A**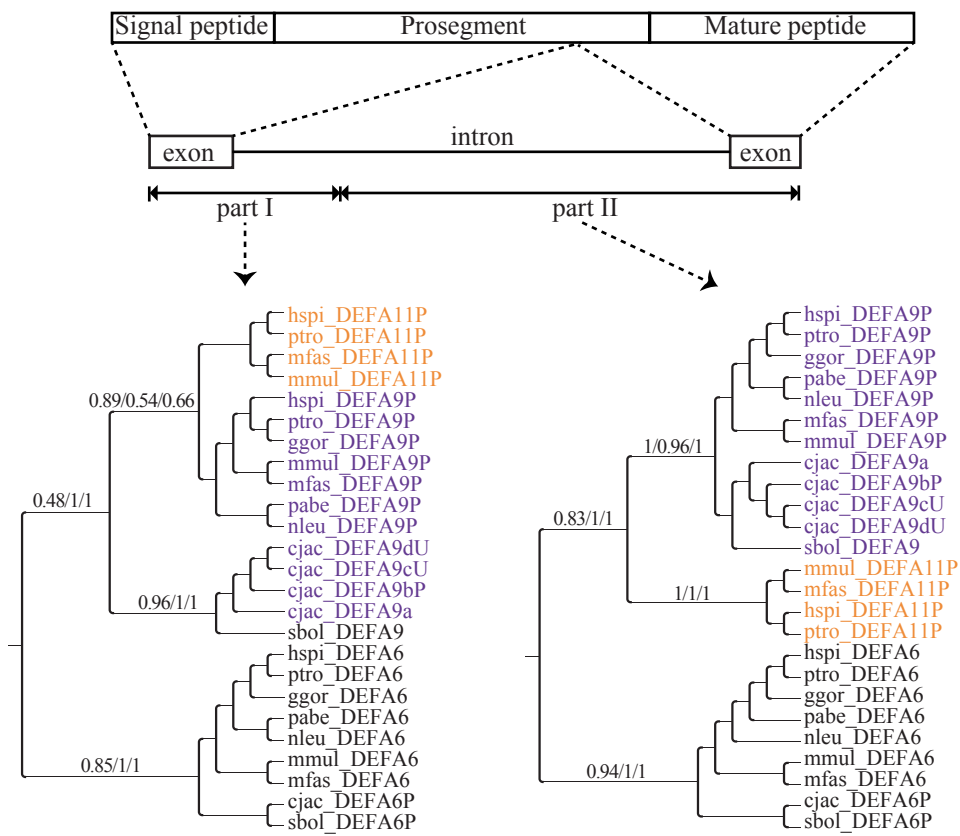**B**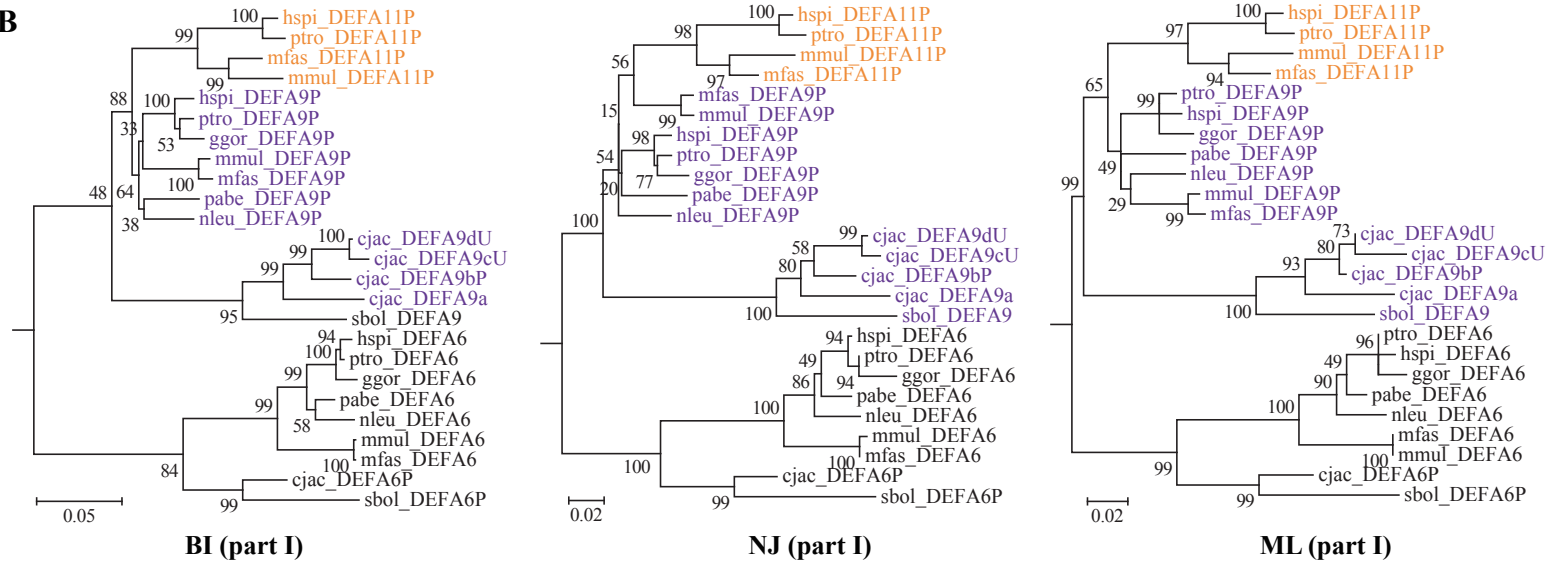**C**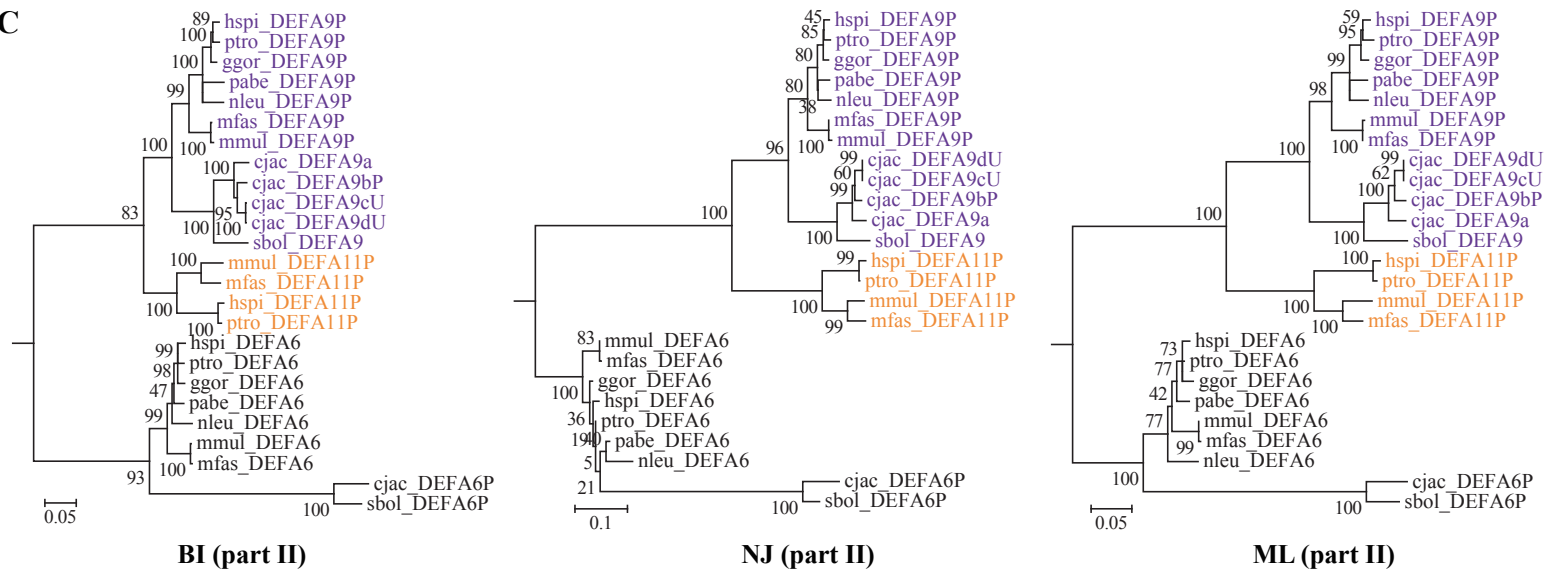

Supplement: Figure S7 — The different phylogenetic relationships of DEFA9/DEFA11 are determined by different parts of the gene. A: Different patterns of sequence similarity are observed before and after a clear boundary in the DEFA9/DEFA11 genes, namely part I and part II. Phylogenetic trees are separately inferred from part I (the tree on the left) and part II (the tree on the right), using the DEFA6 cluster as the outgroup. The combined tree based on part I shows that DEFA11 is duplicated from DEFA9 after the split of New World and Old World monkeys. Whereas, in the combined tree of part II, DEFA11 is the outgroup of all DEFA9 sequences. The BI tree is computed using the GTR + G + I (4 categories) model. The NJ tree is computed using the K2 + G (shape parameter = 1.8 for part I and 3.3 for part II) model. The ML tree is computed using the K2 + G (4 categories) model. B: The BI/NJ/ML trees with branch lengths proportional to the estimated distances inferred from part I. C: The BI/NJ/ML trees inferred from part II. (PDF) [file pone.0097425.s007.pdf]

A

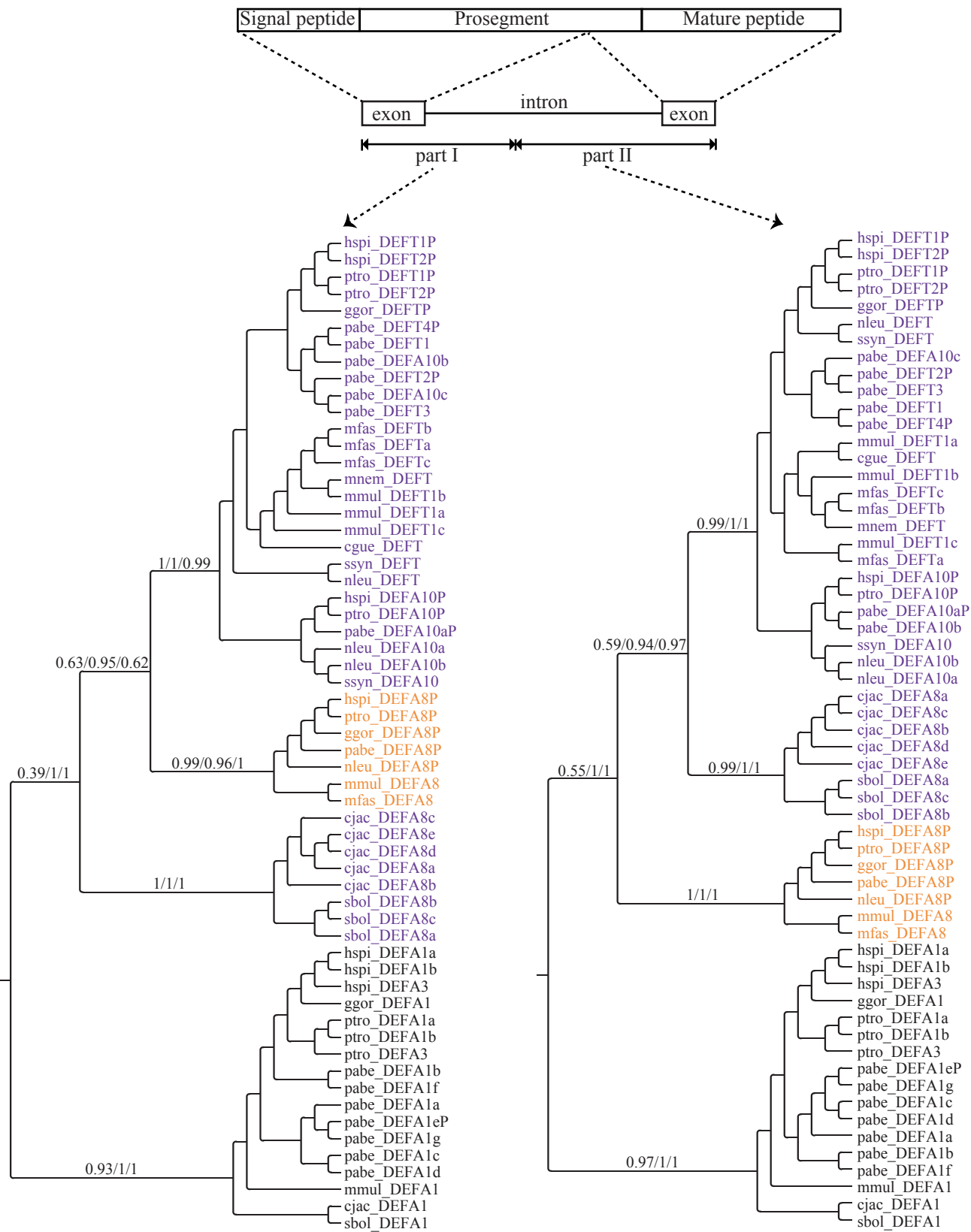

**B**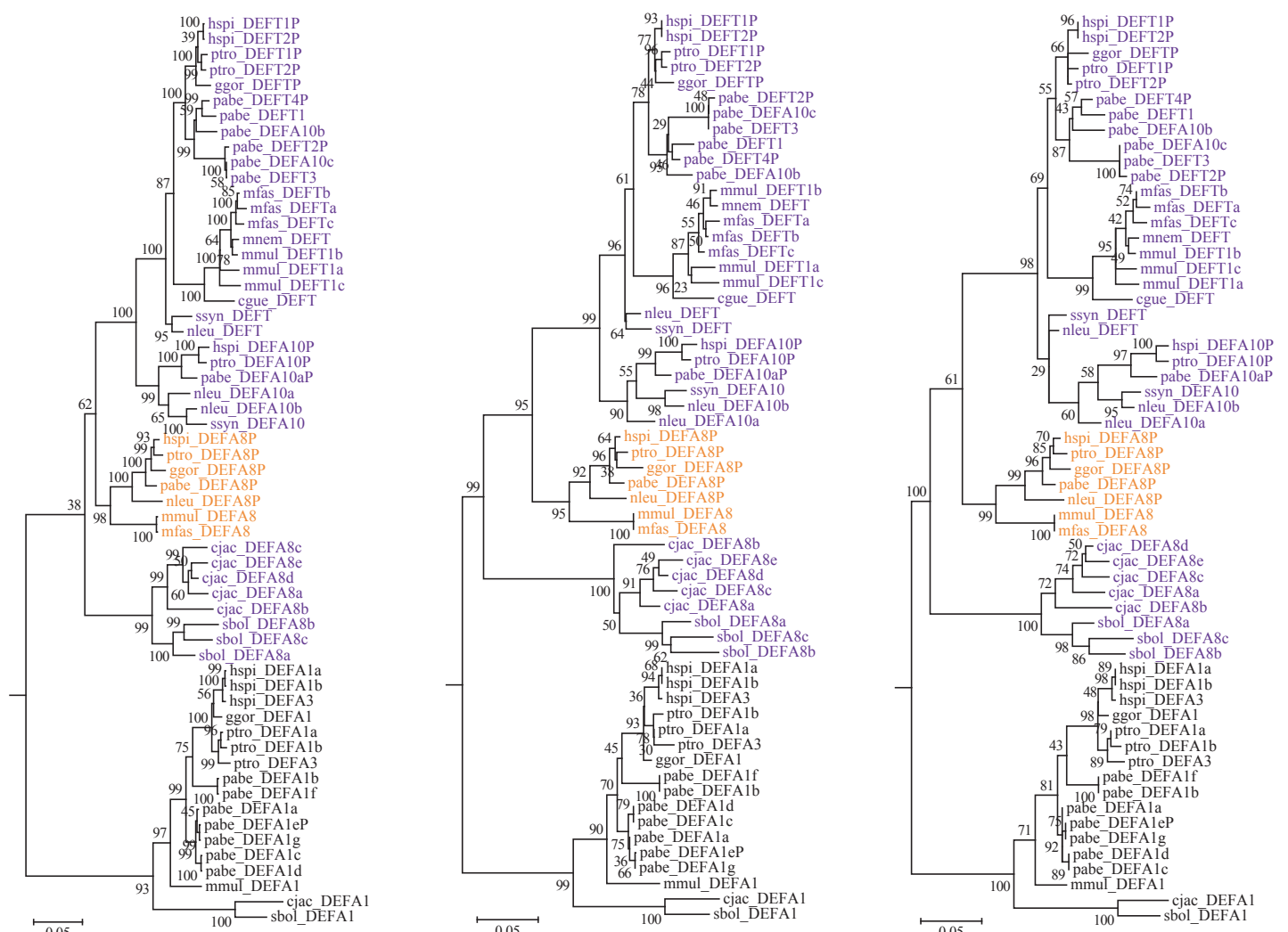**C**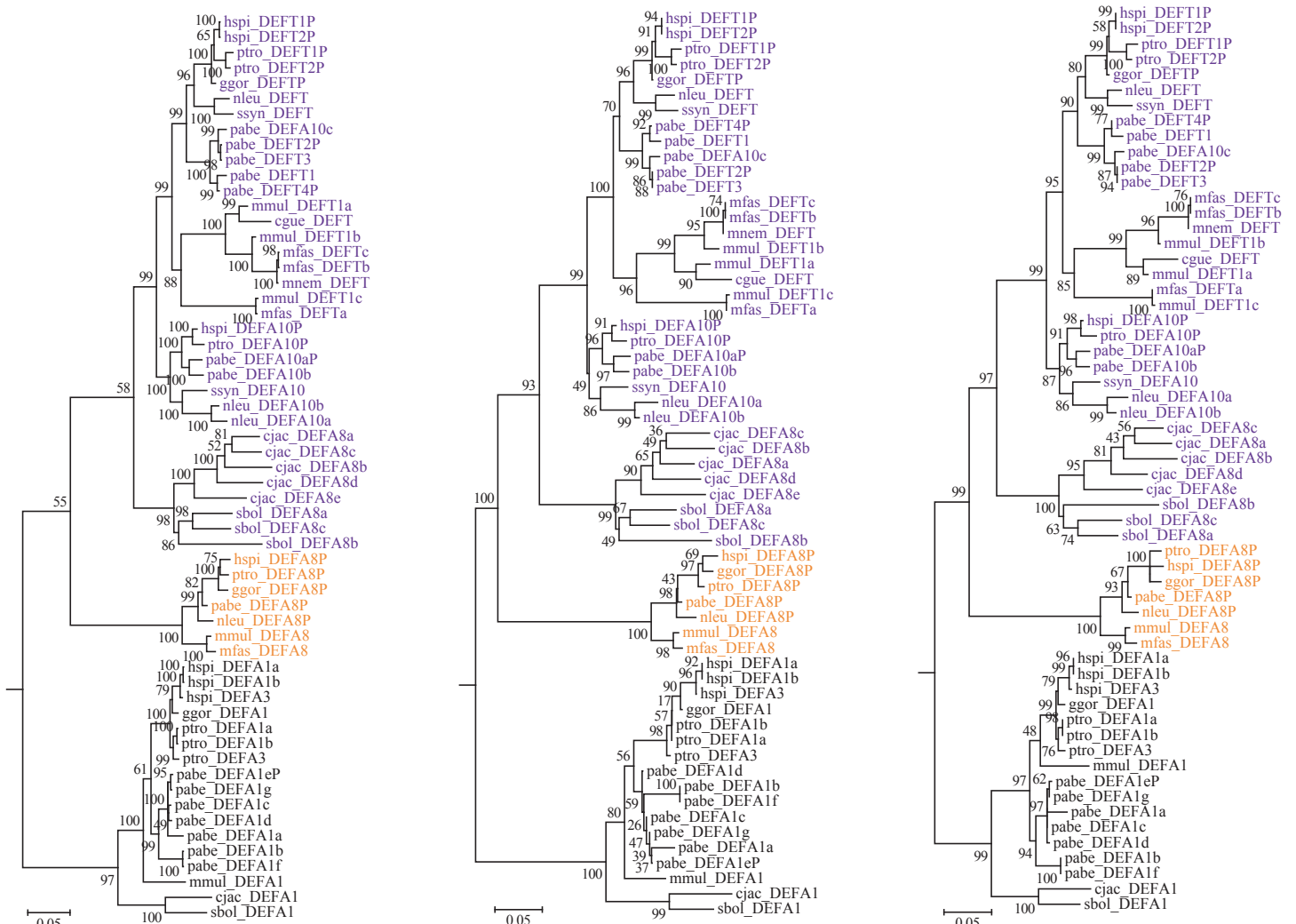

Supplement: Figure S8 — The different phylogenetic positions of hominoid DEFA8 are determined by different parts of the gene. A: Different patterns of sequence similarity are also observed before and after a clear boundary in the DEFA8/DEFA10/DEFT genes, which is different from that of DEFA9/DEFA11 genes. Similarly, phylogenetic trees are separately inferred from part I (the tree on the left) and part II (the tree on the right), using the DEFA1 cluster as the outgroup. In the combined tree inferred from part I, the DEFA8 from Old World monkeys and hominoids clusters together with DEFA10/DEFT. Whereas in the tree inferred from part II, the DEFA8 from Old World monkeys and hominoids is the outgroup in the DEFA8/DEFA10/DEFT cluster. The BI tree is computed using the GTR + G + I (4 categories) model. The NJ tree is computed using the K2 + G (shape parameter = 1.5 for part I and 2.0 for part II) model. The ML tree is computed using the K2 + G (4 categories) model. The sequence pabe_DEFA10c is clustered with DEFT, likely resulting from sequence assembly error. Thus, the sequence pabe_DEFA10c was excluded in following analysis in Figure S9. B: The BI/NJ/ML trees with branch lengths proportional to the estimated distances inferred from part I. C: The BI/NJ/ML trees inferred from part II. (PDF) [file pone.0097425.s008.pdf]

**A**

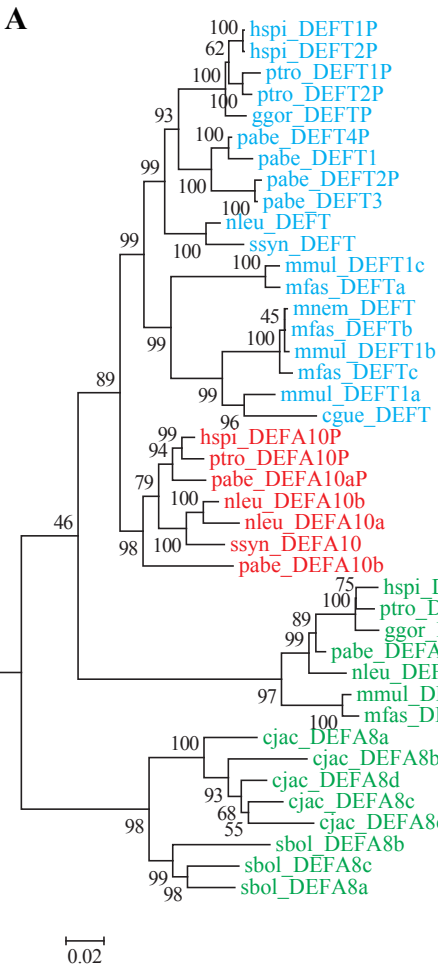

**BI**

**B**

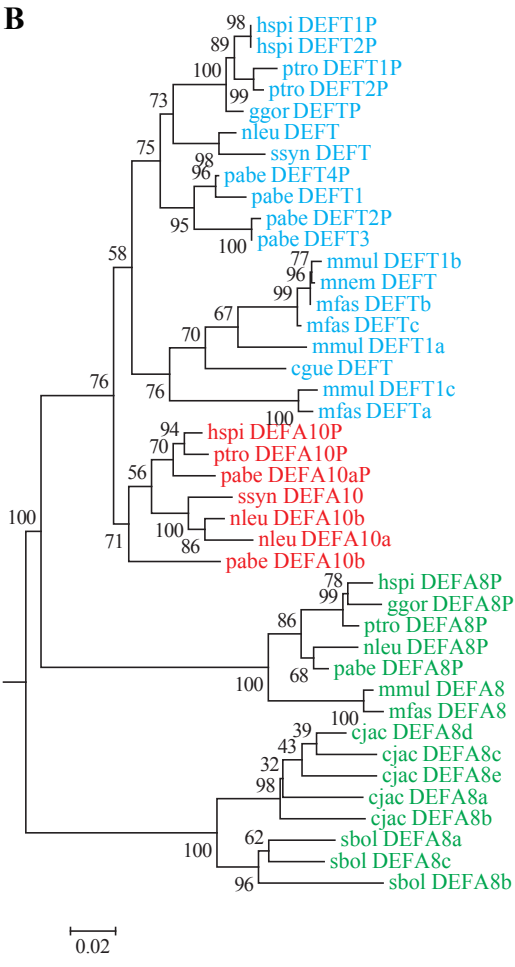

**NJ**

**C**

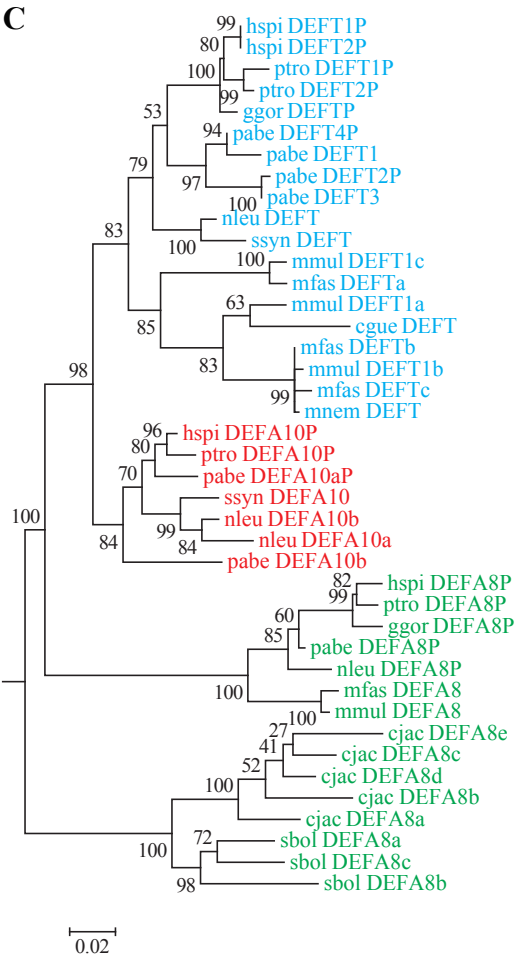

**ML**

Supplement: Figure S9 — Phylogenetic trees constructed using BI (A), NJ (B) and ML (C) methods based on the introns of DEFA8/DEFA10/DEFT . DEFT is found in Old World monkeys and hominoids, whereas DEFA10 is found in hominoids only. The DEFA10 and DEFT are clustered into independent groups and are both derived from the duplication of DEFA8 before the divergence of Old World monkeys and hominoids. But the DEFA10 is lost in the ancestor of Old World monkeys. The BI tree is computed using the GTR + G + I (4 categories) model. The NJ tree is computed using the K2 + G (shape parameter = 1.8) model. The ML tree is computed using the K2 + G (4 categories) model. (PDF) [file pone.0097425.s009.pdf]
