# Supplementary material for: Molecular Evolution of the Primate α-/θ-Defensin Multigene Family
Source: PLoS One. 2014 May 12;9(5):e97425. doi: 10.1371/journal.pone.0097425 (PMC4018336; doi:10.1371/journal.pone.0097425)
Supplement: Table S1 — The repertoires of α-/θ-defensin ( DEFA/DEFT ) genes from primates and treeshrews. (PDF) [file pone.0097425.s010.pdf]

Table S1. The repertoires of α-/θ-defensin (*DEFA/DEFT*) genes from primates and treeshrews

Genus and species	Gene or Pseudogene (P)	Assembly/Contig/Accession ID and Chromosome	Start	End	Strand	Length
Homo sapiens (human)	hspi_DEFA6	GRCh37.p5 chr8	6782340	6783557	-	1218
Homo sapiens (human)	hspi_DEFA4	GRCh37.p5 chr8	6793542	6794421	-	880
Homo sapiens (human)	hspi_DEFA8P	GRCh37.p5 chr8	6808248	6809121	-	874
Homo sapiens (human)	hspi_DEFA9P	GRCh37.p5 chr8	6816811	6817683	-	873
Homo sapiens (human)	hspi_DEFA10P	GRCh37.p5 chr8	6825663	6826635	-	973
Homo sapiens (human)	hspi_DEFA1a	GRCh37.p5 chr8	6835292	6836156	-	865
Homo sapiens (human)	hspi_DEFT1P	GRCh37.p5 chr8	6844833	6845796	-	964
Homo sapiens (human)	hspi_DEFA1b	GRCh37.p5 chr8	6854409	6855273	-	865
Homo sapiens (human)	hspi_DEFT2P	GRCh37.p5 chr8	6863936	6864899	-	964
Homo sapiens (human)	hspi_DEFA3	GRCh37.p5 chr8	6873512	6874372	-	861
Homo sapiens (human)	hspi_DEFA11P	GRCh37.p5 chr8	6886123	6887011	-	889
Homo sapiens (human)	hspi_DEFA7P	GRCh37.p5 chr8	6896093	6896961	-	869
Homo sapiens (human)	hspi_DEFA5	GRCh37.p5 chr8	6912953	6914219	-	1267
Pan troglodytes (chimpanzee)	ptro_DEFA6	gb|AADA01167566.1| chr8	6919082	6920301	-	1220
Pan troglodytes (chimpanzee)	ptro_DEFA4	Pan_troglodytes-2.1.4 chr8	6929894	6930773	-	880
Pan troglodytes (chimpanzee)	ptro_DEFA8P	Pan_troglodytes-2.1.4 chr8	6945241	6946114	-	874
Pan troglodytes (chimpanzee)	ptro_DEFA9P	Pan_troglodytes-2.1.4 chr8	6956971	6957843	-	873
Pan troglodytes (chimpanzee)	ptro_DEFA10P	Pan_troglodytes-2.1.4 chr8	6965813	6966785	-	973
Pan troglodytes (chimpanzee)	ptro_DEFA1a	Pan_troglodytes-2.1.4 chr8	6975344	6976208	-	865
Pan troglodytes (chimpanzee)	ptro_DEFA1b	Pan_troglodytes-2.1.4 chr8	6979548	6980413	-	866
Pan troglodytes (chimpanzee)	ptro_DEFT1P	Pan_troglodytes-2.1.4 chr8	6989086	6990047	-	962
Pan troglodytes (chimpanzee)	ptro_DEFT2P	Pan_troglodytes-2.1.4 chr8	7004600	7005562	-	963
Pan troglodytes (chimpanzee)	ptro_DEFA3	Pan_troglodytes-2.1.4 chr8	7014165	7015029	-	865
Pan troglodytes (chimpanzee)	ptro_DEFA11P	Pan_troglodytes-2.1.4 chr8	7026824	7027708	-	885
Pan troglodytes (chimpanzee)	ptro_DEFA7P	Pan_troglodytes-2.1.4 chr8	7036781	7037649	-	869
Pan troglodytes (chimpanzee)	ptro_DEFA5	Pan_troglodytes-2.1.4 chr8	7053700	7054956	-	1257
Gorilla gorilla (gorilla)	ggor_DEFA6	gorGOR3.1 chr8	6802221	6803431	-	1211
Gorilla gorilla (gorilla)	ggor_DEFA4	gorGOR3.1 chr8	6813117	6813993	-	877
Gorilla gorilla (gorilla)	ggor_DEFA8P	gorGOR3.1 chr8	6827855	6828727	-	873
Gorilla gorilla (gorilla)	ggor_DEFA9P	gorGOR3.1 chr8	6836419	6837286	-	868
Gorilla gorilla (gorilla)	ggor_DEFA1	gorGOR3.1 chr8	6858061	6858925	-	865
Gorilla gorilla (gorilla)	ggor_DEFTP	gorGOR3.1 chr8	6867589	6868552	-	964
Gorilla gorilla (gorilla)	ggor_DEFA5	gorGOR3.1 chr8	6918260	6919514	-	1255
Pongo abelii (Sumatran orangutan)	pabe_DEFA6	AC206038.3 chr8	49291	50523	-	1233
Pongo abelii (Sumatran orangutan)	pabe_DEFA4	AC206038.3 chr8	60288	61167	-	880
Pongo abelii (Sumatran orangutan)	pabe_DEFA8P	AC206038.3 chr8	74929	75800	-	872
Pongo abelii (Sumatran orangutan)	pabe_DEFA9P	AC206038.3 chr8	83490	84364	-	875
Pongo abelii (Sumatran orangutan)	pabe_DEFA10aP	AC206038.3 chr8	92627	93598	-	972
Pongo abelii (Sumatran orangutan)	pabe_DEFA10b	AC206038.3 chr8	102561	103532	-	972
Pongo abelii (Sumatran orangutan)	pabe_DEFA1a	AC206038.3 chr8	115995	116861	-	867
Pongo abelii (Sumatran orangutan)	pabe_DEFT1	AC206038.3 chr8	125503	126471	-	969
Pongo abelii (Sumatran orangutan)	pabe_DEFA1b	AC206038.3 chr8	135104	135968	-	865
Pongo abelii (Sumatran orangutan)	pabe_DEFT2P	AC206038.3 chr8	144608	145576	-	969
Pongo abelii (Sumatran orangutan)	pabe_DEFA1c	AC206038.3 chr8	153825	154689	-	865
Pongo abelii (Sumatran orangutan)	pabe_DEFA10c	AC206038.3 chr8	163331	164300	-	970
Pongo abelii (Sumatran orangutan)	pabe_DEFA1d	AC206038.3 chr8	172558	173422	-	865
Pongo abelii (Sumatran orangutan)	pabe_DEFT3	AC206038.3 chr8	182069	183038	-	970
Pongo abelii (Sumatran orangutan)	pabe_DEFA1eP	AC206038.3 chr8	196567	197430	-	864
Pongo abelii (Sumatran orangutan)	pabe_DEFA1f	AC206038.3 chr8	205118	205982	-	865
Pongo abelii (Sumatran orangutan)	pabe_DEFT4P	AC206038.3 chr8	218858	219826	-	969
Pongo abelii (Sumatran orangutan)	pabe_DEFA1g	AC206038.3 chr8	227327	228191	-	865
Nomascus leucogenys (northern white-cheeked gibbon)	nleu_DEFA6	nomLeu3 chr4	145332766	145333982	+	1217
Nomascus leucogenys (northern white-cheeked gibbon)	nleu_DEFA4	nomLeu3 chr4	145322197	145323078	+	882
Nomascus leucogenys (northern white-cheeked gibbon)	nleu_DEFA8P	nomLeu3 chr4	145307501	145308370	+	870
Nomascus leucogenys (northern white-cheeked gibbon)	nleu_DEFA9P	nomLeu3 chr4	145298344	145299216	+	873
Nomascus leucogenys (northern white-cheeked gibbon)	nleu_DEFA10a	nomLeu3 chr4	145289097	145290064	+	968
Nomascus leucogenys (northern white-cheeked gibbon)	nleu_DEFT	nomLeu3 chr4	145279190	145280161	+	972
Nomascus leucogenys (northern white-cheeked gibbon)	nleu_DEFA10b	nomLeu3 chr4	145271394	145272359	+	966
Nomascus leucogenys (northern white-cheeked gibbon)	nleu_DEFA5	nomLeu3 chr4	145222693	145223940	+	1248
Symphalangus syndactylus (siamang)	ssyn_DEFT	AY128121.1				
Symphalangus syndactylus (siamang)	ssyn_DEFA10	AY128122.1				
Macaca mulatta (macaque)	mmul_DEFA6	rheMac2 chr8	7004643	7005846	-	1204
Macaca mulatta (macaque)	mmul_DEFA4	CABD01041071.1:6324..7200 chr8	7017373	7018248	-	876
Macaca mulatta (macaque)	mmul_DEFA8	rheMac2 chr8	7025712	7026576	-	865
Macaca mulatta (macaque)	mmul_DEFA9P	rheMac2 chr8	7034257	7035131	-	875
Macaca mulatta (macaque)	mmul_DEFT1c	rheMac2 chr8	7050059	7051033	-	975
Macaca mulatta (macaque)	mmul_DEFT1a	rheMac2 chr8	7059969	7060939	-	971
Macaca mulatta (macaque)	mmul_DEFT1b	rheMac2 chr8	7069489	7070462	-	974
Macaca mulatta (macaque)	mmul_DEFA1	rheMac2 chr8	7076982	7077847	-	866
Macaca mulatta (macaque)	mmul_DEFA11P	rheMac2 chr8	7096269	7097148	-	880
Macaca mulatta (macaque)	mmul_DEFA7aP	rheMac2 chr8	7121344	7122209	-	866
Macaca mulatta (macaque)	mmul_DEFA5a	rheMac2 chr8	7129383	7130644	-	1262
Macaca mulatta (macaque)	mmul_DEFA7bUP	rheMac2 chr8	8156206	8157074	-	869
Macaca mulatta (macaque)	mmul_DEFA5bU	rheMac2 chr8	8164259	8165708	-	1450
Macaca mulatta (macaque)	mmul_DEFA5cU	rheMac2 chr8	8172563	8173826	+	1264
Macaca mulatta (macaque)	mmul_DEFA5dU	rheMac2 chr8	8186617	8187878	+	1262
Macaca mulatta (macaque)	mmul_DEFA5eU	rheMac2 chr8	8203388	8204775	+	1388

Table S1 (continued). The repertoires of α-/θ-defensin (*DEFA/DEFT*) genes from primates and treeshrews

Genus and species	Gene or Pseudogene (P)	Assembly/Contig/Accession ID and Chromosome	Start	End	Strand	Length
Macaca fascicularis (crab-eating macaque)	mfas_DEFA6	gb|AEHL01390702.1| chr8	5306	6503	-	1198
Macaca fascicularis (crab-eating macaque)	mfas_DEFA8	gb|AEHL01390704.1| chr8	3792	4656	-	865
Macaca fascicularis (crab-eating macaque)	mfas_DEFA9P	gb|AEHL01390705.1| chr8	4187	5057	-	871
Macaca fascicularis (crab-eating macaque)	mfas_DEFTa	gb|AEHL01390705.1| chr8	19519	20506	-	988
Macaca fascicularis (crab-eating macaque)	mfas_DEFTb	gb|AEHL01390709.1| chr8	8212	9185	-	974
Macaca fascicularis (crab-eating macaque)	mfas_DEFTc	gb|AEHL01390706.1| chr8	1527	2500	-	974
Macaca fascicularis (crab-eating macaque)	mfas_DEFA11P	gb|AEHL01033628.1| chr8	473	1346	+	874
Macaca fascicularis (crab-eating macaque)	mfas_DEFA7P	emb|CAEC01613298.1| chr8	10931	11799	-	869
Macaca fascicularis (crab-eating macaque)	mfas_DEFA5a	emb|CAEC01613250.1| chr8	637	1861	-	1225
Macaca fascicularis (crab-eating macaque)	mfas_DEFA5bP	emb|CAEC01613306.1| chr8	1433	2698	+	1266
Papio anubis (olive baboon)	panu_DEFA5	gb|AC116559.30| chr8	7627	8886	+	1260
Papio anubis (olive baboon)	panu_DEFA7P	gb|AC116559.30| chr8	16062	16929		868
Papio anubis (olive baboon)	panu_DEFTa	NM_001141939.1				
Papio anubis (olive baboon)	panu_DEFTb	NM_001141940.1				
Papio anubis (olive baboon)	panu_DEFTc	NM_001141938.1				
Papio anubis (olive baboon)	panu_DEFTd	NM_001141941.1				
Macaca nemestrina (pig-tailed macaque)	mnem_DEFT	AY128123.1				
Colobus guereza kikuyuensis (eastern black-and-white colobus)	cgue_DEFT	AY128124				
Callithrix jacchus (marmoset)	cjac_DEFA6P	calJac3 chr13	6234467	6235768	-	1302
Callithrix jacchus (marmoset)	cjac_DEFA4	calJac3 chr13	6246148	6247008	-	861
Callithrix jacchus (marmoset)	cjac_DEFA8a	calJac3 chr13	6262870	6263784	-	915
Callithrix jacchus (marmoset)	cjac_DEFA9a	calJac3 chr13	6271720	6272850	-	1131
Callithrix jacchus (marmoset)	cjac_DEFA8b	calJac3 chr13	6282233	6283207	-	975
Callithrix jacchus (marmoset)	cjac_DEFA8c	calJac3 chr13	6297888	6298865	-	978
Callithrix jacchus (marmoset)	cjac_DEFA9bP	calJac3 chr13	6313458	6314900	-	1443
Callithrix jacchus (marmoset)	cjac_DEFA8d	calJac3 chr13	6324765	6325725	-	961
Callithrix jacchus (marmoset)	cjac_DEFA8e	calJac3 chr13	6340090	6341034	-	945
Callithrix jacchus (marmoset)	cjac_DEFA1	calJac3 chr13	6349829	6350671	-	843
Callithrix jacchus (marmoset)	cjac_DEFA5	calJac3 chr13	6382134	6383363	-	1230
Callithrix jacchus (marmoset)	cjac_DEFA9cU	Un_ACFV01191154	2310	3650	-	1341
Callithrix jacchus (marmoset)	cjac_DEFA9dU	Un_ACFV01198749	997	2337	+	1341
Saimiri boliviensis boliviensis (Bolivian squirrel monkey)	sbol_DEFA6P	Broad/saiBol1 JH378324	568357	569851	-	1495
Saimiri boliviensis boliviensis (Bolivian squirrel monkey)	sbol_DEFA4	Broad/saiBol1 JH378324	580399	581274	-	876
Saimiri boliviensis boliviensis (Bolivian squirrel monkey)	sbol_DEFA8a	Broad/saiBol1 JH378324	593624	594517	-	894
Saimiri boliviensis boliviensis (Bolivian squirrel monkey)	sbol_DEFA9	Broad/saiBol1 JH378324	605921	606793	-	873
Saimiri boliviensis boliviensis (Bolivian squirrel monkey)	sbol_DEFA8b	Broad/saiBol1 JH378324	622105	623208	-	1104
Saimiri boliviensis boliviensis (Bolivian squirrel monkey)	sbol_DEFA8c	Broad/saiBol1 JH378324	655945	656893	-	949
Saimiri boliviensis boliviensis (Bolivian squirrel monkey)	sbol_DEFA1	Broad/saiBol1 JH378324	664916	665782	-	867
Saimiri boliviensis boliviensis (Bolivian squirrel monkey)	sbol_DEFA5	Broad/saiBol1 JH378324	695339	696886	-	1548
Microcebus murinus (gray mouse lemur)	mmur_DEFA_a	gb|ABDC01305195.1|	16872	17742	-	871
Microcebus murinus (gray mouse lemur)	mmur_DEFA_b	gb|ABDC01305195.1|	9342	10218	-	877
Microcebus murinus (gray mouse lemur)	mmur_DEFA_c	gb|ABDC01305195.1|	24717	25602	-	886
Microcebus murinus (gray mouse lemur)	mmur_DEFA_dP	gb|ABDC01305195.1|	2247	3123	-	877
Microcebus murinus (gray mouse lemur)	mmur_DEFA_e	gb|ABDC01305192.1|	9933	10779	+	847
Microcebus murinus (gray mouse lemur)	mmur_DEFA_f	gb|ABDC01305193.1|	891	1756	+	866
Microcebus murinus (gray mouse lemur)	mmur_DEFA_gP	gb|ABDC01305191.1|	1612	2476	+	865
Otolemur garnettii (bush baby)	ogar_DEFA_a	gb|AAQR03188273.1|	572	1434	+	863
Otolemur garnettii (bush baby)	ogar_DEFA_b	gb|AAQR03134207.1|	1115	1982	-	868
Otolemur garnettii (bush baby)	ogar_DEFA_c	gb|AAQR03188278.1|	3001	3874	-	874
Otolemur garnettii (bush baby)	ogar_DEFA_d	gb|AAQR03188271.1|	3483	4675	+	1193
Otolemur garnettii (bush baby)	ogar_DEFA_eP	gb|AAQR03188271.1|	11269	12130	+	862
Otolemur garnettii (bush baby)	ogar_DEFA_f	gb|AAQR03188271.1|	19668	20525	+	858
Otolemur garnettii (bush baby)	ogar_DEFA_g	gb|AAQR03188274.1|	1537	2403	+	867
Otolemur garnettii (bush baby)	ogar_DEFA_h	gb|AAQR03188276.1|	1458	2326	-	869
Otolemur garnettii (bush baby)	ogar_DEFA_i	gb|AAQR03188275.1|	3112	3976	-	865
Otolemur garnettii (bush baby)	ogar_DEFA_j	gb|AAQR03134209.1|	3119	3934	-	816
Tarsius syrichta (Philippine tarsier)	tsyr_DEFA_a	gb|ABRT010372935.1|	1672	2543	-	872
Tarsius syrichta (Philippine tarsier)	tsyr_DEFA_bP	gb|ABRT010459708.1|	2275	3146	+	872
Tarsius syrichta (Philippine tarsier)	tsyr_DEFA_cP	gb|ABRT010349769.1|	1642	2492	+	851
Tarsius syrichta (Philippine tarsier)	tsyr_DEFA_d	gb|ABRT010349768.1|	690	1552	+	863
Tarsius syrichta (Philippine tarsier)	tsyr_DEFA_e	gb|ABRT010861540.1|	348	1204	-	857
Tarsius syrichta (Philippine tarsier)	tsyr_DEFA_f	gb|ABRT010331978.1|	2760	3606	-	847
Tarsius syrichta (Philippine tarsier)	tsyr_DEFA_gP	gb|ABRT010486536.1|	2410	3265	+	856
Tarsius syrichta (Philippine tarsier)	tsyr_DEFA_hP	gb|ABRT010668600.1|	66	928	-	863
Tarsius syrichta (Philippine tarsier)	tsyr_DEFA_iP	gb|ABRT010614994.1|	2664	3509	+	846
Tarsius syrichta (Philippine tarsier)	tsyr_DEFA_j	gb|ABRT010623381.1|	689	1583	-	895
Tarsius syrichta (Philippine tarsier)	tsyr_DEFA_k	gb|ABRT010478657.1|	1650	2521	+	872
Tupaia belangeri (northern treeshrew)	tbel_DEFA_aP	gb|AAPY01804345.1|	669	1523	-	855
Tupaia belangeri (northern treeshrew)	tbel_DEFA_b	gb|AAPY01099478.1|	980	1854	+	875
Tupaia belangeri (northern treeshrew)	tbel_DEFA_c	gb|AAPY01477674.1|	11890	12773	+	884
Tupaia belangeri (northern treeshrew)	tbel_DEFA_d	gb|AAPY01509170.1|	6653	7539	+	886
Tupaia belangeri chinensis (Chinese treeshrew)	tbel_DEFA_e	ALAR01162374	25602	26461	+	860
Tupaia belangeri chinensis (Chinese treeshrew)	tbel_DEFA_f	ALAR01162375	3137	4017	-	881
Tupaia belangeri chinensis (Chinese treeshrew)	tbel_DEFA_g	ALAR01162380:1..512,ALAR01162379:4499..4800				
Tupaia belangeri chinensis (Chinese treeshrew)	tbel_DEFA_h	ALAR01162386	18826	19679	+	854
Tupaia belangeri chinensis (Chinese treeshrew)	tbel_DEFA_i	ALAR01162387	6486	7368	-	883
Tupaia belangeri chinensis (Chinese treeshrew)	tbel_DEFA_j	ALAR01198648	1093	1967	+	875
