# Supplementary material for: Molecular Evolution of the Primate α-/θ-Defensin Multigene Family
Source: PLoS One. 2014 May 12;9(5):e97425. doi: 10.1371/journal.pone.0097425 (PMC4018336; doi:10.1371/journal.pone.0097425)
Supplement: Table S2 — Log-likelihood values and parameter estimates for primate DEFA/DEFT genes under site models. Notes: ω = dN/dS; p, number of free parameters; positively selected sites with significance at the 99% level are bold, whereas the remaining sites have significance at the 95% level. The likelihood ratio tests are analyzed by comparing the following pairs of models: M1a-M2a, M7-M8 and M8a-M8. (PDF) [file pone.0097425.s011.pdf]

# Table S2. Log-likelihood values and parameter estimates for primate *DEFA/DEFT* genes under site models

| Cluster | Model | p | lnL | *dN/dS* | Estimates of Parameters | Positively Selected Sites | 2ΔlnL | P value |
|---|---|---|---|---|---|---|---|---|
| DEFT (n=16) | M0: one ratio | 1 | -825.30 | 0.95 | ω | | | |
| | M1a: neutral | 2 | -819.37 | 0.62 | p0=0.38,ω0=0.00; | | | |
| | | | | | p1=0.62,ω1=1.00 | | | |
| | M2a: selection | 3 | -813.08 | 1.06 | p0=0.80,ω0=0.39; | 67I 69R | M1a versus M2a (12.57) | <0.001 |
| | | | | | p1=0.00,ω1=1.00; | | | |
| | | | | | p2=0.20,ω2=3.67 | | | |
| | M7: beta | 2 | -819.38 | 0.61 | p=0.02,q=0.01 | | | |
| | M8: beta and ω | 4 | -813.08 | 1.06 | p0=0.80,p=64.79,q=99.00; | 17Q 50D 67I **69R** | M7 versus M8 (12.60) | 0.002 |
| | | | | | p1=0.20,ω1=3.68 | | M8a versus M8 (12.58) | <0.001 |
| | M8a: beta and ω=1 | 3 | -819.37 | 0.65 | p0=0.38, p=0.01,q=2.65; | | | |
| | | | | | p1= 0.62, ω=1.00 | | | |
| DEFA1 (n=16) | M0: one ratio | 1 | -890.40 | 1.44 | ω | | | |
| | M1a: neutral | 2 | -888.87 | 0.73 | p0=0.27,ω0=0.00; | | | |
| | | | | | p1=0.73,ω1=1.00 | | | |
| | M2a: selection | 3 | -877.66 | 1.90 | p0=0.15,ω0=0.05; | 29V **67Y 74I** | M1a versus M2a (22.42) | <0.001 |
| | | | | | p1=0.69,ω1=1.00; | | | |
| | | | | | p2=0.16,ω2=7.71 | | | |
| | M7: beta | 2 | -889.34 | 0.70 | p=0.01,q=0.01 | | | |
| | M8: beta and ω | 4 | -877.67 | 1.89 | p0=0.83,p=0.96,q=0.26; | 29V **67Y 74I** 86Q | M7 versus M8 (23.33) | <0.001 |
| | | | | | p1=0.17,ω1=7.39 | | M8a versus M8 (22.40) | <0.001 |
| | M8a: beta and ω=1 | 3 | -888.87 | 0.73 | p0=0.27,p=0.01,q=1.80; | | | |
| | | | | | p1=0.73,ω=1.00 | | | |
| DEFA4 (n=8) | M0: one ratio | 1 | -819.95 | 0.73 | ω | | | |
| | M1a: neutral | 2 | -818.22 | 0.66 | p0=0.41,ω0=0.19; | | | |
| | | | | | p1=0.59,ω1=1.00 | | | |
| | M2a: selection | 3 | -816.71 | 0.82 | p0=0.90,ω0=0.52; | | M1a versus M2a (3.02) | 0.082 |
| | | | | | p1=0.00,ω1=1.00; | | | |
| | | | | | p2=0.10,ω2=3.47 | | | |
| | M7: beta | 2 | -818.37 | 0.72 | p=0.05,q=0.02 | | | |
| | M8: beta and ω | 4 | -816.71 | 0.82 | p0=0.90,p=99.00,q=90.87; | | M7 versus M8 (3.32) | 0.190 |
| | | | | | p1=0.10,ω1=3.48 | | M8a versus M8 (3.02) | 0.082 |
| | M8a: beta and ω=1 | 3 | -818.22 | 0.66 | p0=0.41,p=22.71,q=99.00; | | | |
| | | | | | p1=0.59,ω=1.00 | | | |
| DEFA8 (n=15) | M0: one ratio | 1 | -1677.93 | 1.43 | ω | | | |
| | M1a: neutral | 2 | -1645.58 | 0.61 | p0=0.42,ω0=0.07; | | | |
| | | | | | p1=0.58,ω1=1.00 | | | |
| | M2a: selection | 3 | -1585.75 | 1.97 | p0=0.25,ω0=0.00; | **65R 67I 70R** 71G **72I 75L 76L** | M1a versus M2a (119.67) | <0.001 |
| | | | | | p1=0.56,ω1=1.00; | **79R 80Y 82S 84A 85F 87G 92I** | | |
| | | | | | p2=0.19,ω2=7.61 | | | |
| | M7: beta | 2 | -1646.54 | 0.62 | p=0.03,q=0.02 | | | |
| | M8: beta and ω | 4 | -1585.76 | 1.99 | p0=0.81,p=0.01,q=0.01; | **65R 67I 70R** 71G **72I 75L 76L** | M7 versus M8 (121.57) | <0.001 |
| | | | | | p1=0.19,ω1=7.64 | **79R 80Y 82S 84A 85F 87G 92I** | M8a versus M8 (119.66) | <0.001 |
| | M8a: beta and ω=1 | 3 | -1645.59 | 0.61 | p0=0.43,p=8.10,q=99.00; | | | |
| | | | | | p1=0.57,ω=1.00 | | | |
| DEFA5 (n=13) | M0: one ratio | 1 | -1382.57 | 1.51 | ω | | | |
| | M1a: neutral | 2 | -1369.97 | 0.60 | p0=0.47,ω0=0.15; | | | |
| | | | | | p1=0.53,ω1=1.00 | | | |
| | M2a: selection | 3 | -1340.08 | 1.76 | p0=0.33,ω0=0.32; | **68T 70R** 72A **73T 74R** 77L | M1a versus M2a (59.78) | <0.001 |
| | | | | | p1=0.46,ω1=1.00; | **80V 82E 83I 84S** | | |
| | | | | | p2=0.21,ω2=5.65 | | | |
| | M7: beta | 2 | -1372.08 | 0.72 | p=0.04,q=0.01 | | | |
| | M8: beta and ω | 4 | -1340.15 | 1.76 | p0=0.78,p=0.93,q=0.39; | 25R 62A **68T 70R** 72A **73T** | M7 versus M8 (63.85) | <0.001 |
| | | | | | p1=0.22,ω1=5.62 | **74R** 77L **80V 82E 83I 84S** | M8a versus M8 (59.72) | <0.001 |
| | M8a: beta and ω=1 | 3 | -1370.01 | 0.60 | p0=0.47,p=17.94,q=99.00; | | | |
| | | | | | p1=0.53,ω=1.00 | | | |
| DEFA6 (n=7) | M0: one ratio | 1 | -657.09 | 1.50 | ω | | | |
| | M1a: neutral | 2 | -657.74 | 0.97 | p0=0.03,ω0=0.00; | | | |
| | | | | | p1=0.97,ω1=1.00 | | | |
| | M2a: selection | 3 | -656.08 | 1.54 | p0=0.36,ω0=0.00; | | M1a versus M2a (3.33) | 0.068 |
| | | | | | p1=0.00,ω1=1.00; | | | |
| | | | | | p2=0.64,ω2=2.42 | | | |
| | M7: beta | 2 | -657.75 | 1.00 | p=66.66,q=0.01 | | | |
| | M8: beta and ω | 4 | -656.08 | 1.54 | p0=0.36,p=0.01,q=1.43; | | M7 versus M8 (3.35) | 0.187 |
| | | | | | p1=0.64,ω1=2.42 | | M8a versus M8 (3.32) | 0.068 |
| | M8a: beta and ω=1 | 3 | -657.74 | 0.97 | p0=0.03,p=0.01,q=1.03; | | | |
| | | | | | p1=0.97,ω=1.00 | | | |
| DEFA9 (n=4) | M0: one ratio | 1 | -608.40 | 0.65 | ω | | | |
| | M1a: neutral | 2 | -607.70 | 0.68 | p0=0.32,ω0=0.00; | | | |
| | | | | | p1=0.68,ω1=1.00 | | | |
| | M2a: selection | 3 | -606.45 | 1.03 | p0=0.97,ω0=0.59; | | M1a versus M2a (2.51) | 0.113 |
| | | | | | p1=0.00,ω1=1.00; | | | |
| | | | | | p2=0.03,ω2=15.80 | | | |
| | M7: beta | 2 | -607.71 | 0.70 | p=0.01,q=0.01 | | | |
| | M8: beta and ω | 4 | -606.45 | 1.03 | p0=0.97,p=98.40,q=68.88; | | M7 versus M8 (2.52) | 0.284 |
| | | | | | p1=0.03,ω1=15.82 | | M8a versus M8 (2.50) | 0.114 |
| | M8a: beta and ω=1 | 3 | -607.70 | 0.67 | p0=0.36,p=7.11,q=99; | | | |
| | | | | | p1=0.64,ω=1.00 | | | |
| prosimian DEFA clade1 (n=6) | M0: one ratio | 1 | -1184.59 | 0.91 | ω | | | |
| | M1a: neutral | 2 | -1171.40 | 0.69 | p0=0.35,ω0=0.10; | | | |
| | | | | | p1=0.65,ω1=1.00 | | | |
| | M2a: selection | 3 | -1163.34 | 1.28 | p0=0.30,ω0=0.12; | 69I 79R **83V** 87R | M1a versus M2a (16.11) | <0.001 |
| | | | | | p1=0.52,ω1=1.00; | | | |
| | | | | | p2=0.18,ω2=3.97 | | | |
| | M7: beta | 2 | -1171.66 | 0.72 | p=0.04,q=0.02 | | | |
| | M8: beta and ω | 4 | -1163.24 | 1.25 | p0=0.81,p=0.36,q=0.20; | 66H 69I 79R **83V** 87R | M7 versus M8 (16.84) | <0.001 |
| | | | | | p1=0.19,ω1=3.78 | | M8a versus M8 (16.32) | <0.001 |
| | M8a: beta and ω=1 | 3 | -1171.40 | 0.69 | p0=0.35,p=10.71,q=99.00; | | | |
| | | | | | p1=0.65,ω=1.00 | | | |
| prosimian DEFA clade2 (n=14) | M0: one ratio | 1 | -2284.98 | 0.74 | ω | | | |
| | M1a: neutral | 2 | -2204.02 | 0.57 | p0=0.48,ω0=0.11; | | | |
| | | | | | p1=0.52,ω1=1.00 | | | |
| | M2a: selection | 3 | -2165.84 | 1.23 | p0=0.41,ω0=0.12; | **66R 73G** 79Y **87F 90L** | M1a versus M2a (76.36) | <0.001 |
| | | | | | p1=0.46,ω1=1.00; | | | |
| | | | | | p2=0.12,ω2=5.76 | | | |
| | M7: beta | 2 | -2205.46 | 0.55 | p=0.29,q=0.24 | | | |
| | M8: beta and ω | 4 | -2165.76 | 1.20 | p0=0.87,p=0.29,q=0.23; | 37T **66R** 71R **73G** 74F 78T | M7 versus M8 (79.40) | <0.001 |
| | | | | | p1=0.13,ω1=5.42 | **79Y 87F 90L** | M8a versus M8 (72.84) | <0.001 |
| | M8a: beta and ω=1 | 3 | -2202.18 | 0.55 | p0=0.53,p=0.82,q=4.25; | | | |
| | | | | | p1=0.46,ω=1.00 | | | |
