# Supplementary material for: Molecular Evolution of the Primate α-/θ-Defensin Multigene Family
Source: PLoS One. 2014 May 12;9(5):e97425. doi: 10.1371/journal.pone.0097425 (PMC4018336; doi:10.1371/journal.pone.0097425)
Supplement: Table S3 — The average values of Ka, Ks and Ka/Ks from the sliding window analyses for the primate DEFA/DEFT genes and the P values from the bootstrap test. Notes: avg. Ka: average pairwise Ka value; avg. Ks: average pairwise Ks value; avg. Ka/Ks: average pairwise Ka/Ks ratio; Bold number: Ka/Ks significantly >1 (P<0.05); NA: Not available. (PDF) [file pone.0097425.s012.pdf]

Table S3. The average values of Ka, Ks and Ka/Ks from the sliding window analyses for the primate *DEFA/DEFT* genes and the P values from the bootstrap test

**Cluster**	**Sliding window**	**1-10**	**6-15**	**11-20**	**16-25**	**21-30**	**26-35**	**31-40**	**36-45**	**41-50**	**46-55**	**51-60**	**56-65**	**61-70**	**66-75**	**71-80**	**76-85**	**81-90**	**86-93**
	identity	0.90	0.92	0.94	0.90	0.85	0.84	0.83	0.80	0.81	0.79	0.79	0.79	0.81	0.80	0.80	0.80	0.76	0.85
	avg. Ka	0.19	0.17	0.13	0.21	0.34	0.33	0.38	0.46	0.42	0.52	0.59	0.63	0.62	0.70	0.76	0.71	0.96	0.53
DEFA (n=92)	avg. Ks	0.38	0.32	0.33	0.72	0.74	0.52	0.77	0.90	0.76	1.05	1.01	0.77	0.84	0.99	1.06	0.89	0.95	1.06
	avg. Ka/Ks	0.70	0.76	0.58	0.47	0.79	**1.26**	0.94	0.85	0.90	0.69	0.79	**1.46**	**1.48**	**1.15**	**1.03**	**1.33**	**1.59**	0.95
	P value	<0.001	<0.001	<0.001	<0.001	<0.001	<0.001	<0.001	<0.001	<0.001	<0.001	<0.001	<0.001	<0.001	<0.001	0.039	<0.001	<0.001	0.004
	identity	0.97	0.97	0.96	0.93	0.95	0.97	0.98	0.96	0.94	0.95	0.95	0.96	0.93	0.90				
	avg. Ka	0.06	0.07	0.08	0.13	0.19	0.08	0.06	0.12	0.16	0.13	0.10	0.08	0.14	0.19				
DEFT (n=16)	avg. Ks	0.23	0.11	0.14	0.28	0.25	0.20	NA	0.14	0.15	0.26	0.21	NA	0.21	0.35				
	avg. Ka/Ks	0.19	0.43	0.44	0.50	0.37	0.31	NA	0.99	**1.68**	0.50	0.41	NA	0.80	**1.37**				
	P value	<0.001	<0.001	<0.001	<0.001	<0.001	<0.001	NA	0.495	0.017	<0.001	<0.001	NA	0.020	0.030				
	identity	0.98	1.00	0.99	0.99	0.97	0.94	0.92	0.94	0.96	0.96	0.93	0.92	0.95	0.94	0.95	0.96	0.95	0.98
	avg. Ka	0.09	0.05	0.05	0.07	0.07	0.13	0.17	0.20	0.10	0.14	0.18	0.14	0.11	0.13	0.11	0.10	0.10	0.05
DEFA1 (n=16)	avg. Ks	NA	0.11	0.12	0.15	0.14	0.16	0.23	0.19	0.25	0.31	0.36	0.46	NA	0.25	0.21	0.16	0.16	0.23
	avg. Ka/Ks	NA	0.03	0.28	0.48	0.43	0.77	0.81	0.87	0.66	0.65	0.85	0.62	NA	0.62	0.63	0.40	0.47	0.11
	P value	NA	<0.001	<0.001	<0.001	<0.001	<0.001	0.001	0.091	0.002	0.001	0.030	<0.001	NA	<0.001	<0.001	<0.001	<0.001	<0.001
	identity	0.92	0.95	0.96	0.96	0.97	0.93	0.92	0.93	0.97	0.98	0.99	0.97	0.96	0.93	0.91	0.92	0.91	0.96
	avg. Ka	0.15	0.10	0.08	0.06	0.07	0.10	0.14	0.11	0.05	0.06	0.05	0.10	0.14	0.16	0.20	0.16	0.18	0.10
DEFA4 (n=8)	avg. Ks	0.18	0.22	0.44	0.13	0.14	0.16	0.27	0.27	0.21	0.26	0.19	0.20	0.48	0.31	0.30	0.26	0.35	0.23
	avg. Ka/Ks	0.97	0.35	0.18	0.49	0.28	0.48	0.68	0.62	0.14	0.20	0.27	0.51	0.30	0.74	0.37	0.51	0.71	0.46
	P value	0.444	<0.001	<0.001	<0.001	<0.001	<0.001	0.001	<0.001	<0.001	<0.001	<0.001	<0.001	<0.001	0.060	<0.001	<0.001	0.011	<0.001
	identity	0.95	0.94	0.94	0.94	0.91	0.93	0.94	0.88	0.88	0.88	0.89	0.90	0.86	0.83	0.81	0.79	0.78	0.86
	avg. Ka	0.09	0.12	0.11	0.10	0.14	0.10	0.10	0.29	0.27	0.23	0.23	0.25	0.37	0.47	0.60	0.68	0.70	0.38
DEFA8 (n=15)	avg. Ks	0.38	0.23	0.20	0.20	0.29	0.33	0.26	0.40	0.38	0.70	0.38	0.42	0.64	0.47	0.43	0.38	0.47	0.40
	avg. Ka/Ks	0.25	0.58	0.59	0.59	0.56	0.37	0.45	**1.19**	1.11	0.49	0.69	0.85	1.20	**1.64**	**2.11**	**2.82**	**2.18**	**1.57**
	P value	<0.001	<0.001	<0.001	<0.001	<0.001	<0.001	<0.001	0.037	0.157	<0.001	<0.001	0.037	0.073	<0.001	<0.001	<0.001	<0.001	<0.001
	identity	0.96	0.97	0.96	0.96	0.93	0.91	0.90	0.91	0.92	0.92	0.92	0.91	0.90	0.84	0.83	0.83	0.80	0.91
	avg. Ka	0.07	0.13	0.12	0.17	0.19	0.18	0.18	0.14	0.14	0.16	0.15	0.22	0.24	0.40	0.42	0.51	0.67	0.27
DEFA5 (n=13)	avg. Ks	0.17	0.11	0.16	0.15	0.13	0.16	0.21	0.30	0.17	0.15	0.21	0.24	0.24	0.41	0.39	0.39	0.39	0.18
	avg. Ka/Ks	0.34	**1.38**	0.98	**1.66**	**2.87**	0.85	0.61	0.71	0.93	**2.10**	0.86	0.96	**1.61**	**2.07**	**1.63**	**2.40**	**3.74**	**2.37**
	P value	<0.001	0.035	0.432	<0.001	0.001	0.163	<0.001	0.002	0.139	<0.001	0.030	0.324	0.006	<0.001	<0.001	<0.001	<0.001	<0.001
	identity	0.94	0.93	0.97	0.98	0.99	0.97	0.93	0.90	0.94	0.94	0.94	0.97	0.96	0.97	0.97	0.97	0.94	0.95
	avg. Ka	0.11	0.14	0.05	0.04	0.04	0.05	0.13	0.18	0.10	0.12	0.13	0.10	0.12	0.07	0.14	0.20	0.15	0.13
DEFA6 (n=7)	avg. Ks	0.13	0.25	0.13	0.16	0.28	0.26	NA	0.17	0.17	NA	NA	NA	NA	0.19	0.15	NA	NA	NA
	avg. Ka/Ks	1.05	0.65	0.44	0.10	0.05	0.15	NA	0.74	0.42	NA	NA	NA	NA	0.18	0.14	NA	NA	NA
	P value	0.144	<0.001	<0.001	<0.001	<0.001	<0.001	NA	0.162	<0.001	NA	NA	NA	NA	<0.001	<0.001	NA	NA	NA
	identity	0.97	0.97	1.00	0.93	0.91	0.97	0.90	0.87	0.88	0.91	0.93	0.93	0.98	0.98	0.97	0.95	0.95	0.98
	avg. Ka	0.06	0.06	NA	0.13	0.16	0.09	0.19	0.24	0.17	0.15	0.17	0.22	0.08	0.05	0.09	0.14	0.14	0.05
DEFA9 (n=4)	avg. Ks	0.17	0.21	0.19	NA	0.14	0.25	0.57	0.64	0.17	0.30	0.37	0.25	0.14	NA	NA	0.41	1.05	0.93
	avg. Ka/Ks	0.33	0.26	0.00	NA	1.12	0.36	0.33	0.54	1.19	0.60	0.46	0.43	0.40	NA	NA	0.34	0.06	0.03
	P value	<0.001	<0.001	NA	NA	0.298	<0.001	<0.001	<0.001	0.059	<0.001	<0.001	<0.001	<0.001	NA	NA	<0.001	<0.001	<0.001
	identity	0.90	0.94	0.97	0.91	0.84	0.85	0.89	0.87	0.86	0.92	0.89	0.84	0.82	0.84	0.83	0.79	0.76	0.85
prosimian	avg. Ka	0.16	0.11	0.10	0.20	0.42	0.31	0.14	0.21	0.25	0.14	0.22	0.41	0.50	0.44	0.41	0.72	1.08	0.41
DEFA clade1	avg. Ks	0.11	0.29	0.27	0.40	1.36	8.37	0.82	0.51	0.44	0.27	0.34	0.40	0.62	0.49	0.64	0.44	0.75	0.72
(n=6)	avg. Ka/Ks	**2.17**	0.58	0.33	0.65	0.57	0.50	0.29	0.53	0.80	0.60	1.08	**1.64**	1.33	1.20	1.17	**1.73**	**1.63**	0.65
	P value	<0.001	0.003	<0.001	<0.001	<0.001	0.007	<0.001	<0.001	0.108	<0.001	0.401	0.038	0.163	0.216	0.297	0.027	0.003	<0.001
	identity	0.92	0.92	0.95	0.92	0.86	0.86	0.84	0.84	0.83	0.78	0.79	0.79	0.81	0.81	0.81	0.81	0.76	0.85
prosimian	avg. Ka	0.15	0.15	0.08	0.16	0.30	0.26	0.32	0.33	0.34	0.58	0.62	0.54	0.52	0.68	0.76	0.61	1.09	0.67
DEFA clade2	avg. Ks	0.35	0.19	0.17	0.21	0.33	0.51	0.47	0.61	0.90	0.71	0.79	0.64	0.96	1.25	1.14	0.91	0.75	1.07
(n=14)	avg. Ka/Ks	0.53	0.90	0.56	0.86	**1.35**	0.65	0.88	0.83	0.85	1.09	1.04	**1.25**	0.99	0.74	0.77	1.00	**2.53**	0.91
	P value	<0.001	0.058	<0.001	0.017	0.005	<0.001	0.125	0.032	0.066	0.215	0.281	0.004	0.456	<0.001	0.001	0.506	<0.001	0.199
